# Enhancing telesurgical safety with predictive digital twin synchronization: a framework for latency compensation in robotic surgery

**DOI:** 10.1038/s41746-025-02283-w

**Published:** 2026-01-13

**Authors:** Hang Yuan, Junjie Li, Bo Guan, Guangdi Chu, Wei Jiao, Hongzhi Zheng, Xingchi Liu, Jianchang Zhao, Jinhua Li, Jianmin Li, Xuecheng Yang, Haitao Niu

**Affiliations:** 1 Department of Urology, The Affiliated Hospital of Qingdao University, Qingdao, China; 2 Key Laboratory for Mechanism Theory and Equipment Design of Ministry of Education, Tianjin University, Tianjin, China; 3 National Engineering Research Center of Neuromodulation, School of Aerospace Engineering, Tsinghua University, Beijing, China

**Keywords:** Anatomy, Renal cell carcinoma, Mechanical engineering, Clinical trials, Endoscopy

## Abstract

This study addresses the critical challenge of master-slave latency in robot-assisted telesurgery by introducing a Digital Twin Visual Assistance (DTVA) system. DTVA integrates parametric 3D modeling and virtual endoscopic visualization within a tri-layered architecture to enable real-time bidirectional synchronization. The system was evaluated on a geographically distributed robotic platform using programmable latency emulation. Results demonstrated that DTVA maintained spatial precision within 2 mm error under typical conditions and reduced peg-transfer completion time by 13.6% under 900 ms communication latency while lowering operator workload by 27.2%. Clinical validation through teleoperated radical nephrectomy under 300 ms communication latency confirmed feasibility, with all procedures completed successfully without complications and favorable perioperative outcomes. The study establishes DTVA’s capacity to mitigate latency effects and demonstrates preliminary clinical feasibility for telesurgical procedures.

## Introduction

The emergence of robot-assisted telesurgery signifies a paradigm shift in modern healthcare delivery, offering innovative solutions to longstanding challenges in surgical accessibility and precision medicine. This paradigm addresses critical healthcare disparities by enabling surgical expertise dissemination across geographical boundaries, particularly benefiting rural and resource-limited regions through precision intervention capabilities^1–4^. Beyond accessibility improvements, the technology facilitates real-time cross-institutional collaboration, reducing referral system burdens while enhancing emergency response capacities during public health crises. Furthermore, its unique adaptability to extreme environments—from deep-sea operations to extraterrestrial missions—highlights capabilities unattainable in conventional surgical practice^5^. These multidimensional advantages in equitable resource allocation, cost-effective care delivery, and technological convergence collectively position telesurgery as a cornerstone of next-generation telemedicine, driving sustained interdisciplinary innovation toward resilient healthcare ecosystems^4,6–8^.

Despite these advancements, clinical adoption faces persistent technical challenges, with master-slave system latency emerging as a critical bottleneck. Propagation delays between operator commands and robotic responses disrupt visuomotor synchronization—a fundamental requirement for laparoscopic precision^9,10^. This temporal decoupling manifests as dual asynchrony: delayed instrument motion responses paired with misaligned visual feedback, exacerbating targeting inaccuracies and unintended tissue contact risks^11^. Surgeons often compensate through predictive maneuvers based on delayed imaging, inadvertently increasing cognitive load and necessitating conservative techniques that prolong operative durations—a suboptimal tradeoff between safety and efficiency.

While next-generation communication protocols and enhanced control algorithms progressively improve temporal synchronization^12–14^, the inherent network topology constraints of distributed surgical architectures still impose insurmountable inherent latency thresholds. Fundamental physical constraints in signal propagation and computational processing establish theoretical lower bounds that conventional signal optimization strategies cannot overcome, approaching their technical feasibility limits. Although the research paradigm is shifting from pure latency minimization to delay-tolerant intelligent architectures, current solutions exhibit critical human-machine collaboration flaws: over-reliance on autonomous robotic trajectory optimization undermines surgeons’ decision-making dominance, resulting in insufficient temporal decoupling of intraoperative situational awareness and action execution, which severely restricts clinical application^11,15–17^.

Given these fundamental limitations of conventional approaches, digital twin technology emerges as a theoretically viable pathway to mitigate latency challenges by dynamically decoupling operator perception from physical delays. However, despite promising conceptual frameworks, current digital twin implementations face significant barriers to clinical adoption. For instance, the Stereoscopic AR Predictive Display system^18^ utilizes Extended Kalman Filtering and augmented reality for visual prediction, demonstrating reduced task completion times in peg transfer. Yet, its virtual instrument view generation critically depends on initial endoscopic hand-eye calibration using a checkerboard pattern, a step impractical in clinical laparoscopic settings, limiting its clinical translation. Similarly, Bonne et al.‘s digital twin framework employs high-level command abstraction to enhance robustness against network fluctuations for discrete tasks.^19^ Nevertheless, this approach encounters difficulties with latency and manipulation consistency in complex, real-time interactive procedures and requires perfect virtual-real alignment, which is challenging to achieve consistently during actual surgery. Wang et al.‘s “cache-replay” mechanism^20^ provides an innovative solution for disconnection recovery but necessitates full-scene twin reconstruction. This remains unfeasible due to the unresolved technical bottleneck of complex intra-abdominal soft tissue modeling, restricting their system to simple model tasks. Furthermore, Jiang et al.‘s integration of digital twins with Mixed Reality (MR) focuses on improving spatial perception and operator experience via 3D virtual models overlaid on 2D images.^21^ While potentially beneficial for spatial recognition, its design does not target communication delay compensation, and its efficacy under continuous latency gradients remains unexplored. Consequently, while digital twins hold theoretical promise for latency mitigation, the specific technical pathways explored in prior research currently hinder their clinical application.

To overcome these limitations, we propose a Digital Twin Visual Assistance (DTVA) system developed through a distinct technical pathway. This framework system creates a predictive framework that bridges the virtual twin and the physical robot. Its core mechanism proactively simulates the surgeon’s intended actions within the digital twin based on their inputs. This predictive simulation generates immediate visual feedback in the virtual space, effectively counteracting the master-slave latency. By presenting the predicted outcome of actions before the physical robot responds, the system decouples the surgeon’s visual perception from the delayed physical reality. To validate this approach, we rigorously evaluated DTVA across multi-tiered communication latency conditions (5–900 ms) in peg-transfer and suturing tasks, demonstrating significant operational improvements. Critically, DTVA enabled successful completion of remote radical nephrectomy under 300 ms communication latency in realistic constraints—a clinically validated advance for latency-compensated telesurgery. This demonstrates DTVA’s capacity to maintain operational viability in latency-constrained environments.

## Results

### Virtual-physical spatial registration assess of DTVA

**Fig. 1:**
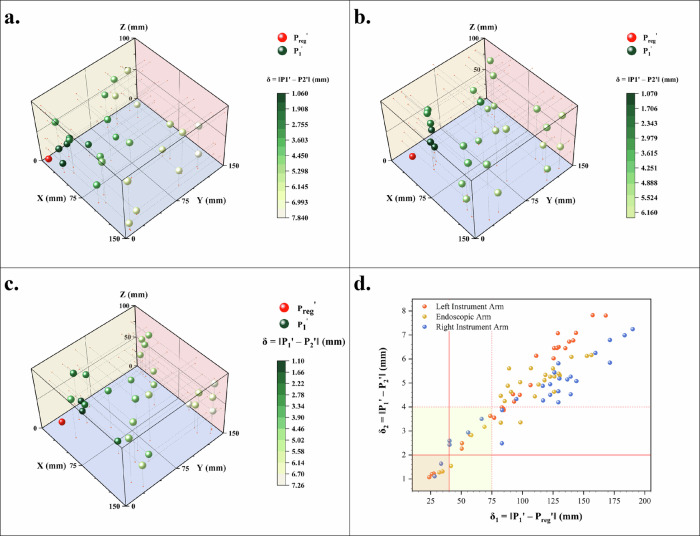
Result for assessing spatial fidelity. Spatial Registration Accuracy of Virtual-Physical Synchronization. End-effector alignment errors (δ₂ = ‖P₁’ - P₂’‖) across robotic arms were quantified as: **a** Left instrument arm (max 7.83 mm @157.35 mm travel), **b** Endoscopic arm (6.16 mm @156.41 mm), **c** Right instrument arm (7.24 mm @190.07 mm). Red spheres denote registration fiducials (P_reg_’), green spheres represent mapped virtual positions (P₁’), with color intensity inversely proportional to δ₂. **d** Virtual-real spatial mismatches (δ₂) versus displacement from registration points (δ₁) for all robotic arms. Showing strong correlation (*r* = 0.919, *p* < 0.001). Subclinical error thresholds (≤2 mm within 4 cm radius) define a clinically viable operational volume.

Experimental evaluation of the virtual-physical synchronization framework identified systematic deviations between the digital twin and physical robotic end-effectors (Fig. 1a–c). Quantitative assessment of spatial mapping accuracy revealed a statistically significant positive correlation (Pearson’s *r* = 0.919, *p* < 0.001) between Virtual-real spatial mismatches (δ_2_) and the spatial displacement from registration fiducials (δ_1_). Distinct error accumulation patterns were observed across the three robotic arms: the left instrument arm exhibited a maximal positional deviation of 7.83 mm at 157.35 mm travel distance, whereas the endoscopic arm demonstrated reduced variability (6.16 mm deviation at 156.41 mm travel). The right instrument arm, operating under extended kinematic demands, displayed a deviation of 7.24 mm at an extreme travel distance of 190.07 mm. These discrepancies arise from the idealized rigid-body assumptions in the digital twin model, diverging from the physical instrument’s real-world behavior under gravitational forces, elastic deformations in cable-driven mechanisms, nonlinear transmission dynamics, and cumulative assembly tolerances.

While the maximum registration error reached 7.83 mm within the 15 × 15 × 10 cm³ workspace, this deviation corresponds to non-physiological diagonal trajectories rarely encountered in clinical laparoscopic practice. As shown in Fig. 1d, δ_2_ remained below 4 mm when the end-effector operated within a 7.5 cm radius of registration points. Restricting the operational radius to 4 cm further reduced errors to ≤2 mm, defining a clinically relevant operational volume of 535.89 cm³ (equivalent to an 8.13 cm edge-length cubic workspace) that adequately supports bilateral instrument arm coordination in typical surgical settings. These results indicate that despite inherent mechanical nonlinearities introducing measurable spatial discrepancies, the system achieves subclinical error thresholds (≤2–4 mm) within constrained operational boundaries, satisfying the spatial precision requirements for routine minimally invasive interventions.

### Latency characterization of the DTVA framework

**Fig. 2:**
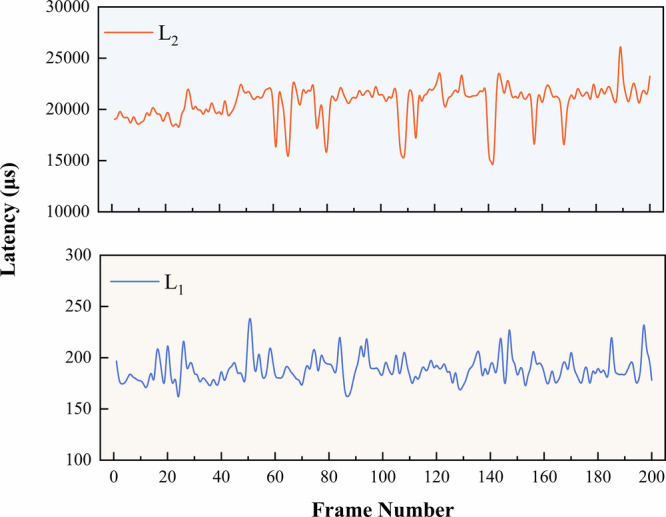
Intrinsic latency characterization of the DTVA framework. The intrinsic latency of DTVA consists of two components: L_1 _+ L_2_. **L₁:** represents the time interval from when the data are received by Qt, processed, and then transmitted to the Unity. **L₂:** refers to the interval from the moment Unity receives the data to the final rendering of the fused display image.

The total intrinsic latency of the DTVA Framework (L₁ + L₂) exhibited millisecond-scale temporal characteristics predominantly determined by L₂. The latency (L_1_) in the Qt framework exhibits microsecond-level stability (188.5 ± 16.2 μs). The latency (L_2_) from Unity data reception to display controls the delay order (20.7 ± 2.08 ms). Aggregate measurements revealed L₂ accounted for 99.1% of the total intrinsic latency (20.86 ± 2.09 ms). When compared to the MicroHand S surgical robot’s inherent 150 ms latency, the DTVA framework’s maximum observed latency (28.3 ms) represents only 18.9% of the baseline system latency. This fundamental disparity confirms the digital twin’s latency profile remains subsumed within existing surgical robot system’s inherent baseline latency budgets, introducing no incremental delay to teleoperation chains. (Fig. 2).

#### Teleoperated peg transfer task operational efficiency

**Fig. 3:**
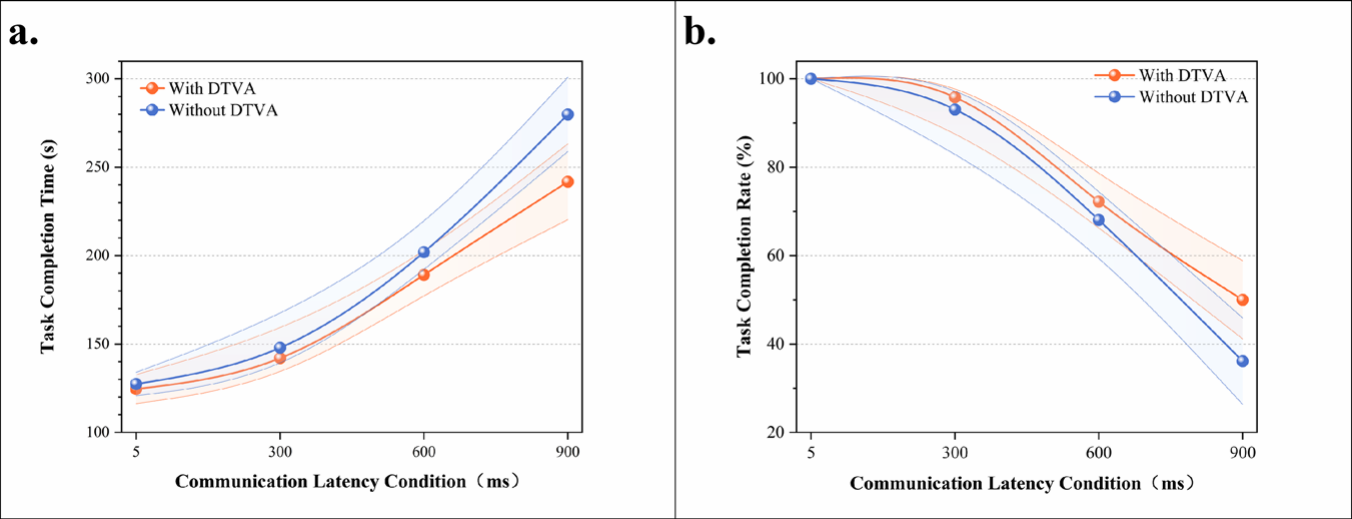
Peg-Transfer Task Operational Efficiency. **a** Task completion time across latency gradients with/without DTVA. Task completion time progressively increased with latency severity, while DTVA attenuated this trend. Data expressed as mean ± SD. The shaded area is formed by connecting error bars representing the standard deviation. **b** Task completion rate across latency gradients with/without DTVA. DTVA implementation improved success rates in a latency-dependent manner. Data expressed as mean ± SD. The shaded area is formed by connecting error bars representing the standard deviation.

Experimental data revealed significant latency-dependent progression in task performance. Under baseline conditions (without DTVA), task completion time exhibited stepwise increases with communication latency. Implementation of the DTVA system significantly reduced completion times across all latency conditions, with improvement magnitudes positively correlated with latency severity (relative reductions: 2.2% at 5 ms, 4.0% at 300 ms, 6.4% at 600 ms, and 13.6% at 900 ms). Notably, even with DTVA assistance, completion time at 900 ms latency remained 19.7% slower than the 600 ms baseline performance (241.80 ms vs. 202.00 ms), demonstrating inherent limitations in compensating for fundamental latency constraints. (Fig. 3a, Table 1).

**Table 1:** Task completion time (s) in teleoperated peg-transfer Task

Communication Latency	DTVA usage		Improvement (%Δ)
	With	Without	
5 ms	124.55 ± 8.21	127.37 ± 6.62	2.21%
300 ms	141.99 ± 14.00	147.91 ± 16.70	4.00%
600 ms	189.16 ± 10.40	202.00 ± 11.13	6.36%
900 ms	241.80 ± 21.44	279.86 ± 20.98	13.60%

Improvement (%Δ) = (Without DTVA - With DTVA)/Without DTVA × 100%. Data expressed as mean ± SD.

A two-way ANOVA confirmed significant main effects of latency magnitude [F(3,64) = 304.905, *p* < 0.001] and DTVA usage [F(1,64) = 18.641, *p* < 0.001], along with a significant interaction effect [F(3,64) = 5.359, *p* = 0.002], accounting for 93.0% of the variance (adjusted R² = 0.930). Simple effects analysis further demonstrated that DTVA implementation showed no statistically significant improvement at 5 ms latency (*p* = 0.684). However, its efficacy became pronounced at higher latencies, with effect sizes escalating proportionally to latency severity. The 900 ms condition yielded the most substantial reduction, where DTVA decreased completion time by 13.6% (241.80 s vs. 279.86 s) and demonstrated significantly stronger compensation effects compared to low-latency environments (interaction *p* < 0.01), highlighting its superior utility in high-latency scenarios. (Table 2).

**Table 2:** Two-way ANOVA results for task completion time in teleoperated peg-transfer task

Source of Variation	Degrees of freedom	Mean Square	F-statistic	P-value
Latency	3	65472.836	304.905	<0.001
DTVA usage	1	4002.888	18.641	<0.001
Latency × DTVA usage	3	1150.76	5.359	0.002

Adjusted R² = 0.930.

Task completion rates provided complementary evidence of latency compensation. Without DTVA, completion rates decayed exponentially with increasing latency. DTVA implementation improved success rates in a latency-dependent manner, achieving a 13.89% absolute improvement at 900 ms (50.00% vs. 36.11%) and progressive enhancements at intermediate latencies (300 ms: +2.77%; 600 ms: +4.16%). Nevertheless, task completion rate under 900 ms latency with DTVA remained 50% lower than the 5 ms baseline, underscoring irreversible performance degradation imposed by fundamental temporal constraints. (Fig. 3b, Table 3).

**Table 3:** Task completion Rate (%) in Teleoperated Peg-Transfer Task

Communication Latency	DTVA usage		Improvement (%Δ)
	With	Without	
5 ms	100.00 ± 0.00	100.00 ± 0.00	0.00%
300 ms	95.83 ± 6.25	93.06 ± 9.08	2.98%
600 ms	72.22 ± 5.51	68.06 ± 6.59	6.11%
900 ms	50.00 ± 8.84	36.11 ± 9.77	38.47%

Data expressed as mean ± SD.

Improvement (%Δ) = (Without DTVA - With DTVA)/Without DTVA × 100%.

### End-effector motion efficiency in teleoperated peg transfer task

**Fig. 4:**
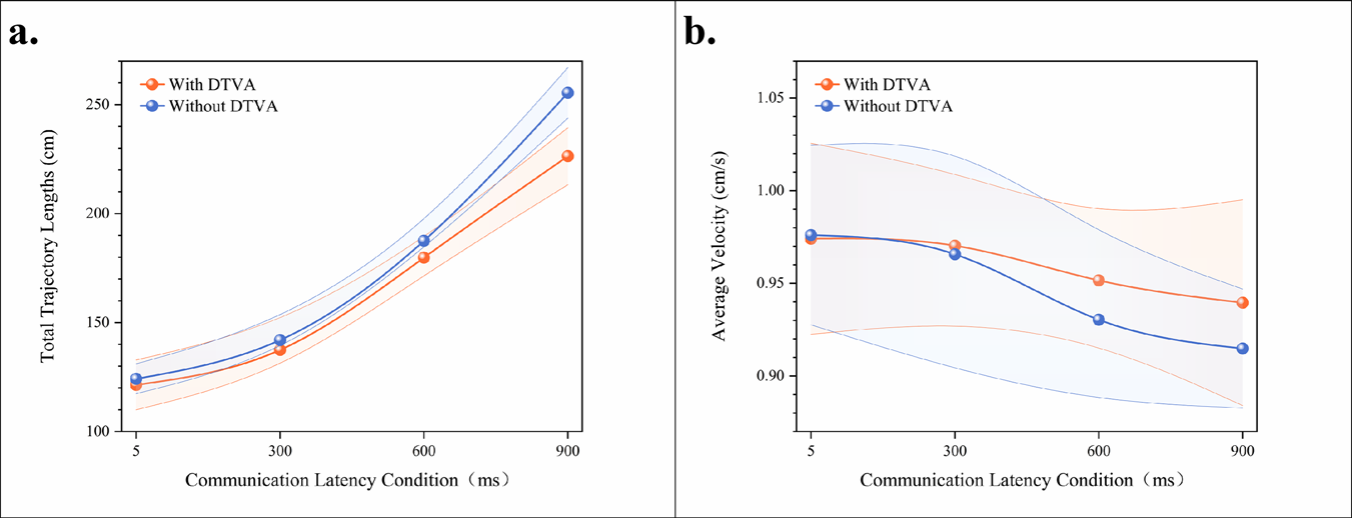
Peg transfer task end-effector motion efficiency. **a** Total trajectory lengths across latency gradients with/without DTVA. Progressive communication latency increased instrument path length, while DTVA implementation significantly optimized path efficiency, with greater improvement at higher latencies. Data expressed as mean ± SD. The shaded area is formed by connecting error bars representing the standard deviation. **b** Average velocity across latency gradients with/without DTVA. Communication latency degraded velocity similarly in both groups, with DTVA yielding no statistically significant improvement despite marginal numerical increases at higher latencies. Data expressed as mean ± SD. The shaded area is formed by connecting error bars representing the standard deviation.

Quantitative trajectory analysis revealed a significant latency-dependent increase in instrument path length, indicating progressive deterioration in movement efficiency. Under control conditions (without DTVA), instrument trajectory length exhibited progressive elongation with increasing communication latency. Implementation of the DTVA system significantly optimized path efficiency across all latency regimes, with improvement magnitude exhibiting proportional escalation to latency severity. (Fig. 4a, Table 4).

**Table 4:** Total Trajectory Lengths (cm) in teleoperated peg-transfer Task

Communication Latency	DTVA usage		Improvement (%Δ)
	With	Without	
5 ms	121.42 ± 11.43	124.21 ± 6.86	2.25%
300 ms	137.48 ± 10.93	141.97 ± 7.71	3.16%
600 ms	179.81 ± 7.63	187.55 ± 4.88	4.13%
900 ms	226.35 ± 13.05	255.44 ± 11.56	11.39%

Data expressed as mean ± SD.

Improvement (%Δ) = (Without DTVA - With DTVA)/Without DTVA × 100%.

A two-way ANOVA confirmed significant main effects of latency magnitude [F(3,64) = 539.400, *p* < 0.001] and DTVA implementation [F(1,64) = 23.590, *p* < 0.001], with a significant interaction effect [F(3,64) = 7.239, *p* < 0.001], accounting for 95.9% of the variance (adjusted R² = 0.959). This substantial trajectory reduction demonstrates DTVA’s capacity to mitigate the adverse effects of latency on operational precision and fluency through predictive visual guidance, particularly under high-latency conditions. The shortened path length directly reflects reduced unnecessary movements and improved accuracy, thereby enhancing task execution efficiency. (Table 5).

**Table 5:** Two-way ANOVA results for total trajectory lengths in teleoperated peg-transfer task

Source of Variation	Degrees of freedom	Mean Square	F-statistic	P-value
Latency	3	50063.082	539.4	<0.001
DTVA usage	1	2189.485	23.59	<0.001
Latency × DTVA usage	3	671.912	7.239	<0.001

Adjusted R² = 0.959.

Complementary velocity analysis demonstrated parallel latency-induced degradation in both groups. Although DTVA yielded numerically higher velocities at ≥300 ms latencies (900 ms: 0.940 vs 0.915, Δ = +0.025), this marginal gain (<3 mm/s equivalent) showed no statistical significance [F(1,64) = 1.208, *p* = 0.276]. The dissociation between significant trajectory optimization and non-significant average velocity changes establishes DTVA’s core compensation mechanism: enhancing spatial efficiency rather than restoring movement speed. This path-optimization strategy—reducing extraneous motion while maintaining operational cadence—exemplifies strategic management of operational boundaries, confirming latency mitigation in this peg transfer paradigm is achieved through movement precision rather than average velocity augmentation. (Fig. 4b, Tables 6, 7).

**Table 6:** Average Velocity (mm/s) in in teleoperated peg-transfer Task

Communication Latency	DTVA usage		Improvement (%Δ)
	With	Without	
5 ms	9.74 ± 0.52	9.76 ± 0.48	−0.20%
300 ms	9.70 ± 0.40	9.66 ± 0.62	0.41%
600 ms	9.52 ± 0.32	9.30 ± 0.44	2.37%
900 ms	9.40 ± 0.56	9.15 ± 0.32	2.73%

Data expressed as mean ± SD.

Improvement (%Δ) = (With DTVA-Without DTVA)/Without DTVA × 100%.

**Table 7:** Two-way ANOVA results for average velocity in teleoperated peg-transfer task

Source of Variation	Degrees of freedom	Mean Square	F-statistic	P-value
Latency	3	0.009	4.137	0.01
DTVA usage	1	0.003	1.208	0.276
Latency × DTVA usage	3	0.001	0.337	0.798

Adjusted R² = 0.097.

### NASA-TLX assessment of operator workload in teleoperated peg transfer task

**Fig. 5:**
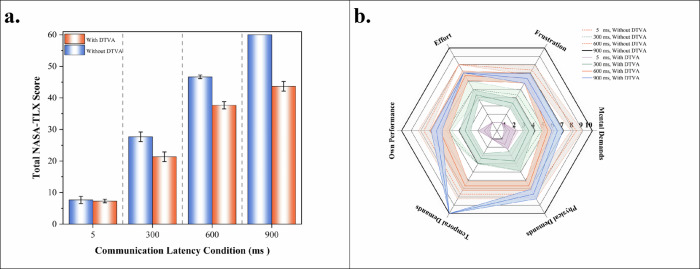
NASA-TLX Assessment of Operator Workload in Teleopetaed Peg Transfer Task. **a** Total NASA-TLX workload scores across latency gradients with/without DTVA. NASA-TLX evaluations demonstrated that the DTVA system significantly redefined the relationship between latency and operator workload, even decoupling high latency from operator workload such that task demands remained manageable under prolonged latency. Data expressed as mean ± SD. **b** Radar plot of NASA-TLX subdimension scores across latency gradients with/without DTVA. The values closer to the center indicate lower task load. Data expressed as mean ± SD. The shaded area is formed by connecting error bars representing the standard deviation.

NASA-TLX evaluations demonstrated that the DTVA system significantly redefined the relationship between latency and operator workload (Fig. 5a and b and Table 8). Under baseline (non-DTVA) conditions, communication latency induced a nonlinear escalation in total workload, peaking at scale’s maximum value of 60 under 900 ms latency, characterized by complete loss of temporal control, frequent operational failures, and extreme frustration. DTVA implementation reduced total workload by 27.2% at 900 ms latency, outperforming even the 600 ms non-DTVA condition, thereby achieving substantive decoupling of high latency from excessive workload.

**Table 8:** NASA-TLX multidimensional workload assessment

Group Dimension	Without DTVA				With DTVA			
	5 ms	300 ms	600 ms	900 ms	5 ms	300 ms	600 ms	900 ms
Mental Demands	1.67 ± 0.58	4.67 ± 0.58	8.33 ± 0.58	10.00 ± 0.00	1.33 ± 0.58	3.33 ± 0.58	5.33 ± 0.58	6.33 ± 0.58
Physical Demands	1.67 ± 0.58	4.33 ± 0.58	7.66 ± 0.58	10.00 ± 0.00	1.67 ± 0.58	3.67 ± 1.15	6.67 ± 0.58	7.67 ± 0.58
Temporal Demands	1.00 ± 0.00	4.00 ± 0.00	7.66 ± 0.58	10.00 ± 0.00	1.00 ± 0.00	3.33 ± 0.58	6.67 ± 0.58	10.00 ± 0.00
Own Performance	1.33 ± 0.58	5.33 ± 0.58	7.66 ± 0.58	10.00 ± 0.00	1.33 ± 0.58	3.33 ± 0.58	6.00 ± 0.58	6.33 ± 0.58
Effort	1.00 ± 0.00	5.00 ± 0.00	8.00 ± 0.00	10.00 ± 0.00	1.00 ± 0.00	4.33 ± 0.58	6.67 ± 0.58	7.00 ± 0.00
Frustration	1.00 ± 0.00	4.33 ± 0.58	7.33 ± 0.58	10.00 ± 0.00	1.00 ± 0.00	3.33 ± 0.58	6.33 ± 0.58	6.33 ± 0.58
Total Score	7.67 ± 1.15	27.67 ± 1.53	46.67 ± 0.58	60 ± 0.00	7.33 ± 0.58	21.33 ± 1.53	37.67 ± 1.15	43.67 ± 1.53

Two-way ANOVA quantified the joint effects of latency and DTVA, revealing significant main effects of latency magnitude (F(3,16) = 1784.086, *p* < 0.001) and DTVA intervention [F(1,16) = 297.290, *p* < 0.001], with a strong interaction effect [F(3,16) = 51.097, *p* < 0.001]. Simple effects analysis identified threshold-enhanced characteristics: when latency exceeded 200 ms, DTVA’s workload reduction exhibited nonlinear amplification, with the absolute reduction at 900 ms (Δ = 16.33) being 1.8-fold greater than at 600 ms (Δ = 9.00). *(*Table 9*).*

**Table 9:** Two-way ANOVA results for NASA-TLX Score in teleoperated peg-transfer task

Source of Variation	Degrees of freedom	Mean Square	F-statistic	P-value
Latency	3	2304.444	1784.086	<0.001
DTVA usage	1	384	297.29	<0.001
Latency × DTVA usage	3	66	51.097	<0.001

Adjusted R² = 0.996.

**Fig. 6:**
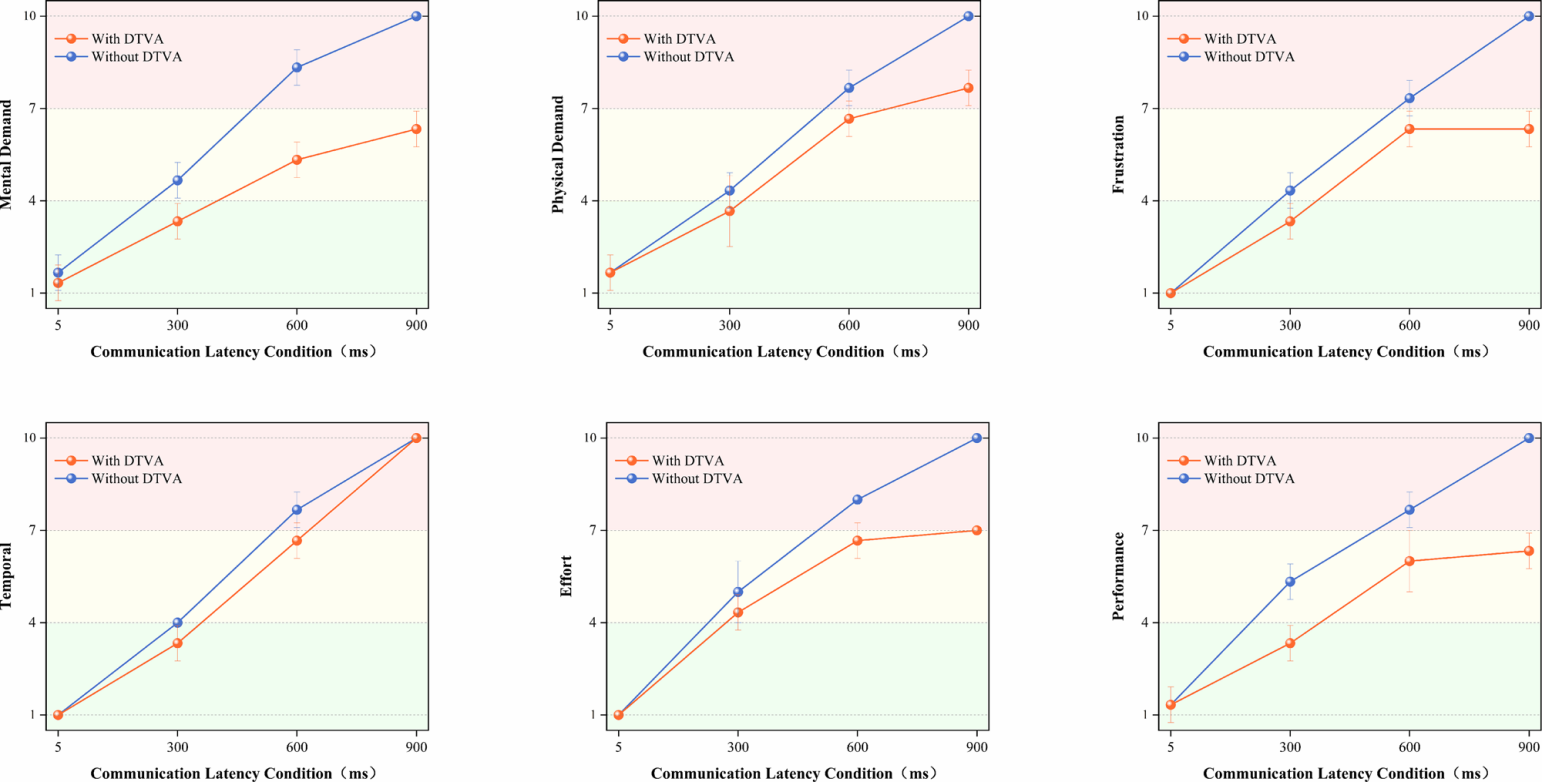
NASA-TLX assessment of operator workload in teleopetaed peg transfer task. NASA-TLX Subdimensional Workload Profiling across latency gradients with/without DTVA. Subdimensional analysis demonstrated DTVA’s latency-dependent workload regulation. At 300 ms, Mental Demand and Effort decreased, remaining within comfort thresholds. At 600 ms, all subdimensions stayed below compensatory thresholds. Under 900 ms, DTVA maintained subcritical Frustration and optimized performance despite extreme temporal demand. Data expressed as mean ± SD.

Subdimensional analysis revealed distinct patterns in DTVA’s dynamic compensation mechanisms across varying latency conditions. Under minimal latency (5 ms), inter-dimensional workload scores exhibited an approaching Ideal Operating State (1 points), suggesting limited requirement for systemic intervention. At 300 ms latency, DTVA demonstrated significant reductions in critical workload dimensions, constraining Mental Demand to 3.33 ± 0.58 and Effort to 4.33 ± 0.58 compared to baseline operations without DTVA, thereby maintaining operator workload within the Comfort Operating Threshold (<4 points). The system demonstrated enhanced regulatory capacity at 600 ms latency, constraining all subdimensions below the Compensatory Threshold (7 points), effectively preventing workload escalation into unstable operational zones. Under extreme 900 ms latency, DTVA achieved multidimensional optimization despite prolonged operational durations: Mental and Frustration remained subcritical, though Physical Demand persisted near compensatory limits (7.67 ± 0.58 vs. 10.00 ± 0.00). Notably, temporal pressure (Temporal Demand) reached the Load Limit State (10 point) under 150 s task constraints. However, DTVA preserved operational continuity through performance rhythm optimization (ΔPerformance = +3.67 points), demonstrating its capacity to transcend conventional latency-workload limitations via human-machine collaborative restructuring strategies. (Figs. 5b, 6*).*

### Operational efficiency in teleoperated suturing task

**Fig. 7:**
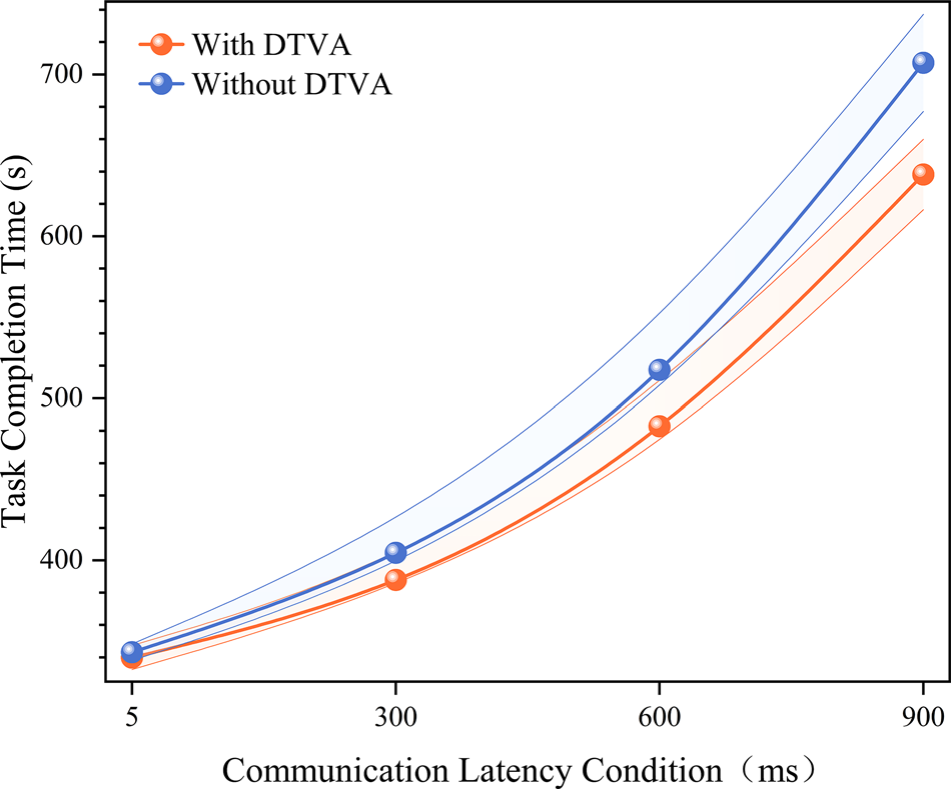
Task completion time in teleoperated Suturing Task. Communication latency progressively increased operation time under control conditions (Without DTVA). Implementation of the DTVA system significantly reduced completion times across all latency levels, with greater relative improvements observed at higher latencies. The shaded area is formed by connecting error bars representing the standard deviation.

Experimental data revealed a significant latency-dependent increase in operation time, indicating progressive deterioration in task execution efficiency. Under control conditions (without DTVA), completion time exhibited progressive elongation with increasing communication latency. Implementation of the DTVA system significantly optimized temporal efficiency across all latency regimes, with improvement magnitude exhibiting proportional escalation to latency severity. Notably, at 600 ms latency, DTVA reduced operation time by 34.65 s compared to baseline (482.82 s vs. 517.47 s), equivalent to 30.7% of the latency-induced time increase observed between 300 ms and 600 ms conditions without DTVA (Δ = 112.82 s). (Fig. 7 and Table 10).

**Table 10:** Task completion time (s) in teleoperated Suturing Task

Communication Latency	DTVA usage		Improvement (%Δ)
	With	Without	
5 ms	339.89 ± 7.40	343.19 ± 5.13	0.96%
300 ms	387.84 ± 7.78	404.65 ± 13.35	4.15%
600 ms	482.82 ± 19.85	517.47 ± 22.42	6.70%
900 ms	638.18 ± 21.76	707.17 ± 30.04	9.76%

Data expressed as mean ± SD.

Improvement (%Δ) = (Without DTVA - With DTVA)/Without DTVA × 100%.

A two-way ANOVA confirmed significant main effects of latency magnitude [F(3,16) = 393.113, *p* < 0.001] and DTVA implementation [F(1,16) = 17.722, *p* = 0.001], with a significant interaction effect [F(3,16) = 3.743, *p* = 0.033], accounting for 98.1% of the variance (adjusted R² = 0.981). This substantial time reduction demonstrates DTVA’s capacity to mitigate the adverse effects of latency on procedural efficiency through predictive visual guidance, particularly under high-latency conditions. The shortened operation time directly reflects streamlined task execution and reduced corrective maneuvers, thereby enhancing surgical workflow efficiency. (Table 11).

**Table 11:** Two-way ANOVA results for Task completion time in teleoperated Suturing Task

Source of Variation	Degrees of freedom	Mean Square	F-statistic	P-value
Latency	3	127383.821	393.113	<0.001
DTVA usage	1	5742.464	17.722	0.001
Latency × DTVA usage	3	1212.734	3.743	0.033

Adjusted R² = 0.981.

### End-effector motion efficiency in teleoperated suturing task

**Fig. 8:**
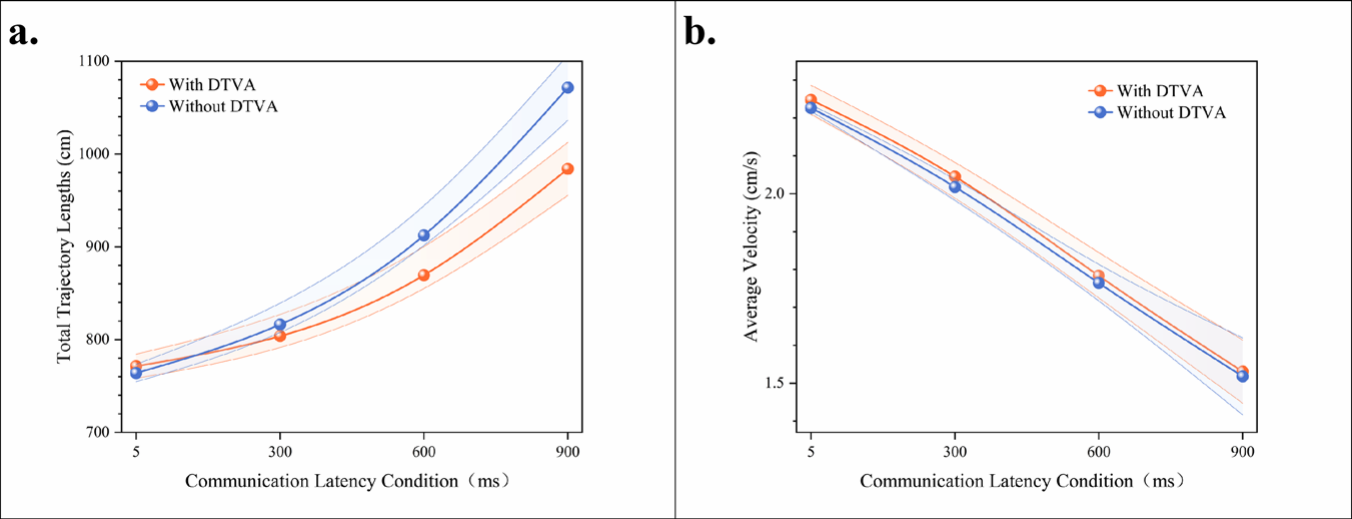
Suturing task end-effector motion efficiency. **a** Communication latency progressively increased instrument path length without DTVA. DTVA significantly reduced trajectory length across all latency levels, with greater relative improvement at higher delays. Consistent with peg-transfer tasks. The shaded area is formed by connecting error bars representing the standard deviation. **b** Average velocity decreased significantly with increasing communication latency in both control and DTVA conditions. Unlike its effect on trajectory optimization, DTVA implementation failed to significantly alter average velocity across all latency levels. Notably, the suturing task exhibited more pronounced velocity reduction than peg-transfer tasks as latency increased.

Quantitative trajectory analysis revealed a significant latency-dependent increase in instrument path length, indicating progressive deterioration in movement efficiency. Under control conditions (without DTVA), instrument trajectory length exhibited progressive elongation with increasing communication latency. Implementation of the DTVA system significantly optimized path efficiency across latency regimes, with improvement magnitude exhibiting proportional escalation to latency severity. Notably, at 600 ms latency, DTVA reduced trajectory length by 42.86 cm compared to control (869.49 cm vs. 912.35 cm), equivalent to 44.6% of the latency-induced path increase observed between 300 ms and 600 ms conditions without DTVA. (Fig. 8a and Table 12).

**Table 12:** Total trajectory lengths (cm) in teleoperated suturing task

Communication Latency	DTVA usage		Improvement (%Δ)
	With	Without	
5 ms	771.31 ± 13.02	763.98 ± 9.32	−0.96%
300 ms	803.77 ± 17.90	816.22 ± 16.47	1.53%
600 ms	869.49 ± 22.21	912.35 ± 19.18	4.70%
900 ms	983.98 ± 28.49	1071.46 ± 35.12	8.16%

Data expressed as mean ± SD.

Improvement (%Δ) = (Without DTVA - With DTVA)/Without DTVA × 100%.

A two-way ANOVA confirmed significant main effects of latency magnitude [F(3,16) = 167.603, *p* < 0.001] and DTVA implementation [F(1,16) = 14.665, *p* = 0.001], with a significant interaction effect [F(3,16) = 5.446, *p* = 0.009], accounting for 95.8% of the variance (adjusted R² = 0.958). This substantial trajectory reduction demonstrates DTVA’s capacity to mitigate latency-induced spatial inefficiency through predictive visual guidance, particularly under high-latency conditions. The optimized path length directly reflects reduced unnecessary movements and improved instrument control, thereby enhancing surgical precision. (Table 13)*.*

**Table 13:** Two-way ANOVA results for Total Trajectory Lengths in teleoperated Suturing Task

Source of Variation	Degrees of freedom	Mean Square	F-statistic	P-value
Latency	3	78648.86	167.603	<0.001
DTVA usage	1	6881.707	14.665	0.001
Latency × DTVA usage	3	2555.579	5.446	0.009

Adjusted R² = 0.958.

Complementary velocity profiling identified progressive latency-induced deceleration across experimental conditions, with control group velocities declining from 22.26 mm/s (5 ms) to 15.18 cm/s (900 ms), mirroring the DTVA group’s speed reduction from 22.70 mm/s to 15.43 mm/s. While DTVA consistently demonstrated numerically higher velocities at all latency tiers, statistical analysis confirmed these gains were non-significant. Critically, the non-significant latency × DTVA interaction [F(3,16) = 0.060, *p* = 0.980] established that DTVA failed to alter the fundamental latency-velocity decay relationship. This velocity preservation occurred despite concurrent, statistically robust trajectory optimization, reinforcing DTVA’s compensation paradigm: enhancing spatial efficiency through path refinement without kinematically accelerating movement execution. (Fig. 8b and Tables 14, 15).

**Table 14:** Average velocity (mm/s) in Teleoperated suturing task

Communication Latency	DTVA usage		Improvement (%Δ)
	With	Without	
5 ms	22.70 ± 0.47	22.26 ± 0.09	1.98%
300 ms	20.73 ± 0.49	20.18 ± 0.30	2.73%
600 ms	18.03 ± 0.74	17.64 ± 0.40	2.21%
900 ms	15.43 ± 0.82	15.18 ± 1.01	1.65%

Data expressed as mean ± SD.

Improvement (%Δ) = (With DTVA-Without DTVA)/Without DTVA × 100%.

**Table 15:** Two-way ANOVA results for Average Velocity in teleoperated Suturing Task

Source of Variation	Degrees of freedom	Mean Square	F-statistic	P-value
Latency	3	0.584	158.155	<0.001
DTVA usage	1	0.01	2.683	0.121
Latency × DTVA usage	3	0	0.06	0.98

Adjusted R² = 0.953.

### Suture quality assessment in Teleoperated Suturing Task

Suturing performance was systematically evaluated under varying communication latency conditions (5 ms, 300 ms, 600 ms, 900 ms) with or without DTVA assistance. Analysis of the comprehensive suture quality index (Q_c_) revealed significant main effects for both DTVA usage (F(1,16) = 5.492, *p* = 0.032) and latency (F(3,16) = 19.571, *p* < 0.001). The statistical model explained 72.0% of Qc variance (adjusted R² = 0.720). At 5 ms latency, Q_c_ scores were comparable between DTVA and non-DTVA conditions. Progressive latency increases differentially impacted performance: at 300 ms, non-DTVA trials exhibited Q_c_ degradation relative to DTVA-assisted operations; at 600 ms, tissue tearing incidents emerged in non-DTVA trials (2/15 stitches) but remained absent with DTVA (0/15). Under 900 ms latency, non-DTVA trials demonstrated substantial performance deterioration characterized by frequent tissue tearing (7/15 stitches) and compromised suture quality, whereas DTVA implementation maintained functional task completion with significantly reduced tearing incidence (3/15 stitches) and improved Q_c_ scores. (Tables 16, 17*).*

**Table 16:** Suture quality assessment in Teleoperated Suturing Task

Group Quality	With DTVA				Without DTVA			
	5 ms	300 ms	600 ms	900 ms	5 ms	300 ms	600 ms	900 ms
Q_1_	3.00 ± 0.00	2.00 ± 0.00	1.00 ± 1.00	0.33 ± 0.58	3.00 ± 0.00	2.00 ± 0.00	1.00 ± 1.00	0.33 ± 0.58
Q_2_	2.33 ± 0.58	2.00 ± 0.00	1.33 ± 0.58	0.67 ± 0.58	2.33 ± 0.58	2.00 ± 0.00	1.33 ± 1.16	0.33 ± 0.58
Q_3_	3.00 ± 0.00	2.33 ± 0.58	1.00 ± 1.00	1.00 ± 1.73	3.00 ± 0.00	2.33 ± 0.58	1.00 ± 1.00	0.33 ± 0.58
Q_c_	8.33 ± 0.58	8.00 ± 0.00	5.67 ± 0.58	3.00 ± 2.65	8.33 ± 0.58	6.33 ± 0.58	3.33 ± 2.89	1.00 ± 1.73
Q_s_	0/15	0/15	0/15	3/15	0/15	0/15	2/15	7/15

Data expressed as mean ± SD.

**Table 17:** Two-way ANOVA results for comprehensive suture quality (Qc) in teleoperated Suturing Task

Source of Variation	Degrees of freedom	Mean Square	F-statistic	P-value
Latency	3	48.111	19.571	<0.001
DTVA usage	1	13.5	5.492	0.032
Latency × DTVA usage	3	1.611	0.655	0.591

Adjusted R² = 0.720.

### Clinical outcomes of the telesurgery under DTVA

**Fig. 9:**
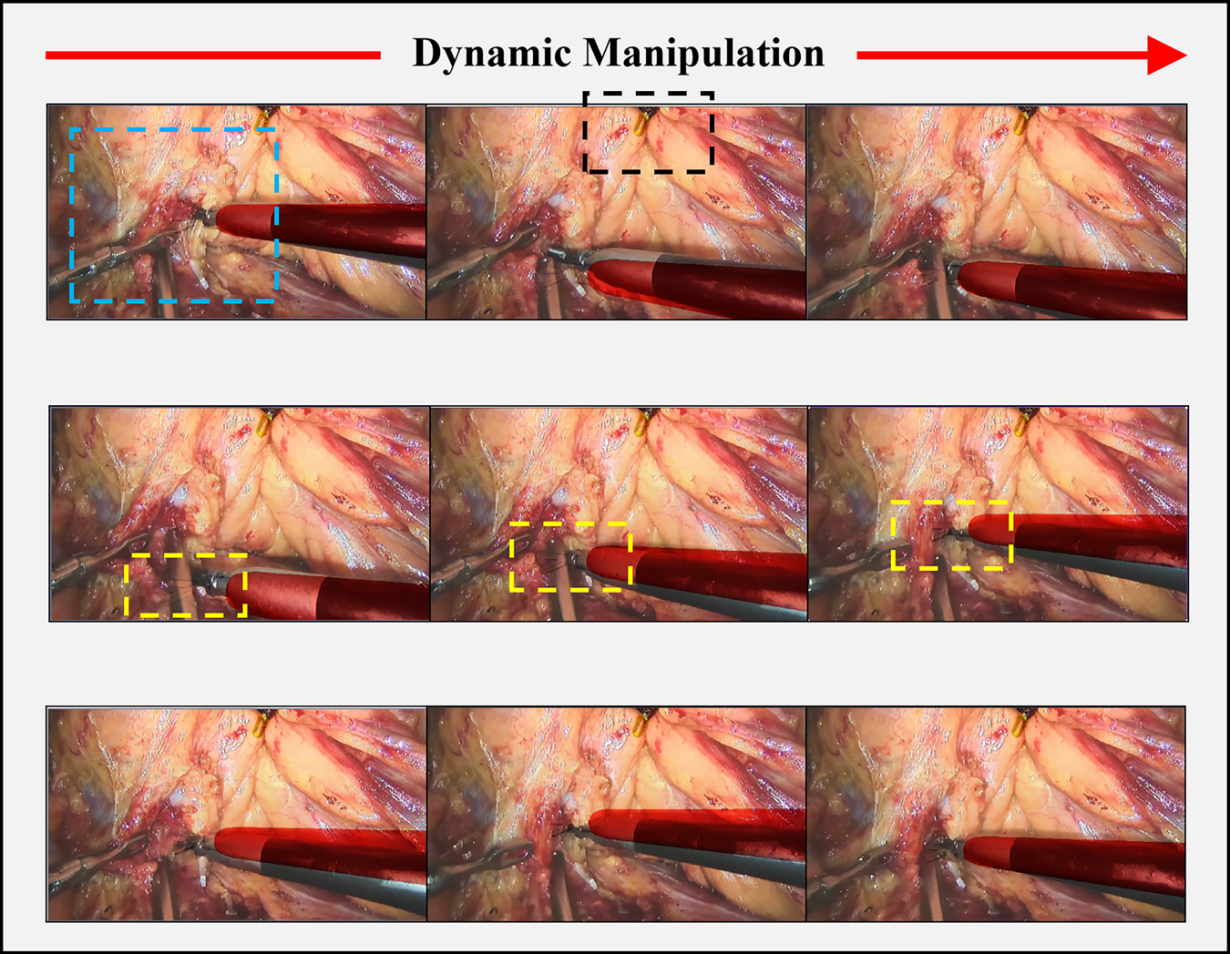
Real-time field-of-view fusion and intraoperative visualization during Renal Hilar Dissection. Blue dashed box: Anatomical localization of the renal hilum. Black dashed box: Left robotic grasper (physical instrument) lifting tissue, with its digital twin fully overlapping the real instrument due to static state. Yellow dashed box: Virtual tip of the right robotic instrument, dynamically superimposed on the real endoscopic view. The virtual instrument tip remains unobstructed despite physical occlusions, providing continuous spatial guidance critical for safe teleoperation.

Between January 2025 and March 2025, 3 patients (2 males, 66.7%; 1 female, 33.3%) were enrolled in this study, with a median age of 57 years (range: 51–58). 2 patients (66.7%) were categorized as ASA class II, while the remaining were ASA class I. All procedures achieved complete tumor resection with a median operative time of 115 min (range: 105–127) and median estimated blood loss of 100 mL (range: 40–120). No intraoperative complications or conversions to alternative surgical approaches occurred. Postoperative recovery was uneventful, with a median 24-h visual analog scale (VAS) pain score of 3 (range: 2–4). All cases were classified as Clavien-Dindo grade I at discharge, indicating minor deviations from the normal postoperative course without requiring pharmacological or surgical interventions. Follow-up CT imaging and laboratory tests confirmed the absence of residual tumors or surgical sequelae in all patients. All patients recovered well during the 3-month postoperative follow-up. (*Detailed demographic and perioperative parameters are summarized in* Table 18, Fig. 9).

**Table 18:** Patient characteristics, clinical outcomes, and system performance metrics

Parameter	Result
**Perioperative**	
BMI (kg/m^2^), Median (range)	24.16 (21.33–25.76)
Age (yr), Median (range)	57 (51–58)
Gender (n), Male/ Female	2 /1
Previous Pelvic/abdominal surgery, n (%)	0 (0%)
Tumor side (n), Right/ Left	1 /2
R.E.N.A.L score, Median (range)	8 (7–10)
ASA Classification, n (%)	
Ⅰ	1 (33.3%)
Ⅱ	2 (66.7%)
**Intraoperative**	
Total latency (ms)	450
Communication latency (ms)	300
Robot processing latency (ms)	150
DTVA latency (ms), Mean (SD)	20.86 (2.09)
Master-slave distance (km)	209.2
Operative time (min), Median (range)	115 (105–127)
Estimated blood loss (ml), Median (range)	100 (40–120)
**Postoperative**	
VAS for 24 h, Median (range)	3 (2–4)
Clavien–Dindo Grade, n (%)	
Ⅰ	3(100%)
≥Ⅱ	0
Postoperative hospital stay (d), Median (range)	3(3–4)
Pathological stage, *n* (%)	
T_1b_ N_0_ M_0_	3(100%)

## Discussion

The emergence of telesurgery represents a fundamental transformation in global healthcare delivery, offering unprecedented opportunities to democratize surgical expertise by transcending geographic and socioeconomic barriers^1–3^. While traditional telemedicine primarily focuses on diagnostic and consultative services, the integration of robotic systems with advanced communication technologies enables true surgical decentralization^2,4,6^. This technological convergence holds particular significance for resource-limited regions facing critical shortages of surgical specialists. Our study addresses the fundamental challenge of temporal dissociation in master-slave systems—a barrier that has hindered the clinical translation of telesurgery concepts for over two decades. Persistent delays between operator actions and robotic responses create neurocognitive dissonance, disrupting the visuomotor integration essential for millimeter-level precision in procedures like laparoscopy. Conventional mitigation strategies, ranging from network protocol optimization to predictive control algorithms, are approaching their technical limits due to insurmountable physical constraints. Our DTVA framework achieves theoretical advancement by redefining latency—not as a technical flaw to be eliminated, but as an environmental parameter adaptable through human-centered systems. By implementing digital modeling and real-time visual fusion for virtual-physical synchronization, DTVA creates a “visuomotor delay bridge” through predictive virtual feedback. This allows surgeons to interact with a predictive simulation that anticipates instrument trajectories ahead of physical reality, enhancing cognitive adaptation through augmented predictive cues. Such prospective visualization establishes a cognitive buffer layer against temporal asynchrony, effectively decoupling human perception from time delays while preserving human agency in decision-making.

To comprehensively validate the performance of the theoretical framework, systematic quantification of the DTVA system was conducted through latency characterization and virtual-physical alignment assessment. Latency quantification within the hierarchical digital twin framework confirmed that the integrated DTVA does not interfere with existing telesurgical operations. Specifically, the system’s maximum total latency (28.3 ms) constituted merely 18.9% of the master robotic platform’s inherent baseline latency (150 ms), with its tiered architecture effectively preventing additional latency generation. This fundamental latency advantage enables the digital twin instruments to maintain predictive motion display ahead of physical operations. Crucially, such predictive capability provides continuous visual pre-alert functionality even under theoretically ideal latency-free communication conditions. Virtual-physical spatial registration assessment quantified system accuracy under static conditions (post-motion stabilization), confirming sufficient registration precision (<2 mm error in the 4 cm core workspace). In dynamic operation, DTVA’s core operational mechanism employs predictive instrument display to compensate latency, which inherently positions the virtual instrument ahead of the moving real instrument. This temporal offset—fundamental to latency compensation—results in predictable registration differences during motion. Consequently, static registration metrics characterize baseline precision but do not reflect dynamic operational conditions. Critically, within this functional paradigm, subsequent experiments decisively demonstrated that despite both this dynamic misalignment and the known static precision limitations, DTVA effectively counters latency effects: In controlled peg transfer and suturing tasks across 5–900 ms communication latency conditions, the system consistently enhanced operator performance compared to non-DTVA controls. Clinical validation further confirmed that radical nephrectomy under 300 ms communication latency remains feasible within the established precision framework. These collective findings indicate that within the static registration accuracy constraints established experimentally, procedural success in delayed environments is enabled by DTVA’s real-time dynamic guidance mechanism. This mechanism leverages the predictive motion trajectories of latency-free virtual instruments to define operational boundaries.

The peg-transfer experiments demonstrate that DTVA’s effectiveness increases with communication latency severity. DTVA progressively reduced task completion time and shortened instrument paths without altering average movement speed, confirming its efficiency improvement stems from reducing unnecessary motions through predictive guidance—not by accelerating average velocity. NASA-TLX workload reduction also exhibited threshold-dependent amplification. Remarkably, under 900 ms latency, DTVA achieved 27.2% total workload reduction, with surgeons experiencing lower workload at 900 ms using DTVA than at 600 ms without it. This decoupling phenomenon highlights DTVA’s optimization strategy focused on cognitive resource allocation rather than mechanical efficiency. By offloading spatial planning to virtual interfaces, DTVA relieves surgeons of visuomotor coordination burdens. This cognitive reallocation delivers dual advantages: enhanced human-robot collaboration (enabling better control with fewer corrections and less frustration), and liberated capacity for surgical decision-making—particularly critical in complex procedures where decision fatigue accumulates nonlinearly with operative duration. Of particular note is DTVA’s minimal performance improvement under near-ideal 5 ms latency conditions, suggesting diminishing returns in low-latency environments. Conversely, its utility progressively increases when latency exceeds 300 ms, revealing an adaptive compensation mechanism: when disrupted sensorimotor loops impair natural operation, the system transitions from redundant assistance to essential cognitive scaffolding. While 900 ms latency caused 19.7% slower task completion versus the 600 ms baseline, DTVA’s 13.6% improvement demonstrates significant mitigation of temporal constraints. Crucially, DTVA maintained manageable workload levels under extreme communication latency, confirming its role in preserving operational viability under simulated latency extremes rather than eliminating latency entirely.

The ex vivo porcine renal suturing experiments further validated DTVA’s latency-dependent efficacy and operational optimization. Without significantly altering average movement velocity, DTVA achieved dual spatiotemporal efficiency improvements by reducing task completion time and optimizing instrument trajectories. These enhancements proportionally intensified with latency severity, corroborating core findings from peg-transfer studies. Clinically most significant were the dual-dimensional advancements in suturing quality: Technically, the comprehensive quality index (Q_c_) demonstrated markedly improved suture integrity under communication latencies exceeding 300 ms. Regarding safety, tissue tearing incidents were substantially reduced—eliminated entirely at 600 ms latency (0/15 stitches vs. 2/15 control) and decreased to 42.9% of control levels at 900 ms (3/15 vs. 7/15). This dual breakthrough in technical precision and safety assurance during delicate renal tissue manipulation confirms DTVA’s capacity to enhance instrument control accuracy in low-error-tolerance complex tasks. By reducing corrective maneuvers through predictive guidance, DTVA directly strengthens human-robot collaborative efficacy under communication latency.

The clinical validation of DTVA under realistic constraints demonstrated successful completion of procedures under specified latency. All three radical nephrectomies assisted by DTVA were completed under a constant 300 ms communication delay, with all cases achieving Clavien-Dindo grade <III outcomes—a confirmation of the system’s safety and feasibility under such latency conditions. DTVA’s virtual instrument ghosting technique maintained spatial awareness while providing safety warnings in latency-affected environments. During renal hilum dissection in narrow anatomical regions where instrument mobility is restricted, the confined surgical spaces inherently defined the threshold requirements for virtual-physical registration accuracy. Furthermore, the virtual camera system ensures uninterrupted visualization of instrument tips when superimposed on endoscopic views, enabling continuous positional tracking even amid physical obstructions. This capability establishes a critical safety mechanism by maintaining the surgeon’s spatial awareness during complex maneuvers where traditional visualization would be compromised by overlapping anatomical structures. As validated in Fig. 9, this unobstructed tracking significantly enhances real-time orientation in challenging scenarios. Notably, this advantage extends beyond high-latency teleoperation to benefit conventional robot-assisted laparoscopy, demonstrating its versatility across surgical platforms.

While this study demonstrates the clinical feasibility of DTVA in latency compensation, several critical limitations must be acknowledged to guide future research. First, the parametric physical modeling based on rigid-body kinematics overlooked critical mechanical variances, including elastic deformations, cumulative angular deviations in cable-driven systems, and spatial mismatches induced by tissue contact forces. As evidenced by our spatial fidelity results (maximum δ_2_ = 7.83 mm), these simplifications restrict effective operational volumes from encompassing multiple anatomical quadrants, necessitating recalibration after endoscope repositioning—a process disrupting procedural continuity. Second, our network emulation framework employed constant latency (5–900 ms) with zero packet loss (packet loss rate <0.001%), failing to replicate real-world challenges such as delay jitter, burst packet loss risks, or QoS heterogeneity from multi-hop international transmissions. These factors could induce temporal reference drift and compromise end-to-end synchronization, particularly in extreme scenarios, where system robustness remains unverified. Third, the NDI-based spatial registration accuracy assessment method itself introduced inherent uncertainties; while NDI systems are highly accurate, distinguishing error sources—whether attributable to NDI measurement limitations or rigid-body modeling assumptions—proved challenging, complicating algorithmic refinements to optimize twin-instrument motion patterns. Fourth, while the original NASA-TLX scale is extensively validated and widely adopted globally, we introduced behavioral anchors at critical points to enhance scoring objectivity and interpretability—addressing known limitations of subjective magnitude estimation in surgical settings. Although such adaptations are methodologically common^22^, our modified version (retaining the six core dimensions) lacked formal validation. The absence of reliability testing and construct validity verification introduces uncertainty in workload quantification accuracy. Fifth, while the ex vivo suturing quantified technical performance via Qc metrics, they remain proxy indicators divorced from physiological validation, this ex vivo assessment failed to capture the paramount clinical endpoint of hemostatic security—the prevention of intraoperative or delayed postoperative hemorrhage through reliably sealed incisions. Consequently, DTVA’s efficacy in achieving suture integrity that translates to hemorrhage prevention requires confirmation in survival animal models. Finally, both experimental and clinical validations suffered from restricted operator diversity: peg-transfer and suturing tasks were conducted by limited operator across limited repetitions, while urology-specific clinical procedures involved only one surgeon operating on a small cohort (*n* = 3) under exclusively 300 ms latency. The combination of limited operator diversity and small sample sizes may constrain generalizability across varying levels of operator expertise and different surgical contexts. Addressing these limitations will require integrating deformable modeling, dynamic network emulation, multi-source error attribution frameworks, and expanded task protocols across broader surgical specialties.

Despite these limitations, this work demonstrates that digital twin technology provides an effective approach to latency management in telesurgery. By providing predictive spatial guidance, DTVA enables surgeons to maintain operative precision under high-latency conditions previously considered prohibitive—validated through both controlled experiments and real-world procedures. This approach shifts the paradigm from combating latency to strategically compensating for its cognitive impact, transforming communication latencies from operational barriers into manageable constraints. The framework’s unique capacity to maintain procedural continuity where conventional teleoperation falters unlocks transformative potential for deploying surgical expertise into geographically isolated and resource-constrained environments devoid of reliable, low-latency communication. This capability is critically enabling for mission-critical scenarios where low-latency connectivity is compromised or absent, such as cross-regional disaster relief operations coordinating care across vast, damaged landscapes; maritime medical support aboard vessels undertaking extended oceanic voyages; remote medical outposts in austere settings far from tertiary centers; and battlefield casualty care demanding immediate, expert intervention under hostile conditions. By decoupling complex surgical guidance from the stringent demands of real-time, high-bandwidth networking, this technology could expand the reach of specialized surgical care, offering a promising strategy in addressing healthcare disparities and delivering life-saving interventions where conventional telemedicine fails, thereby expanding the boundaries of accessible, high-acuity medicine across the globe. Future development towards such resilient systems will involve DTVA operating as a key component within a broader technological ecosystem, synergizing with advances in areas including secure communications^8^ to mitigate a wider spectrum of telesurgical risks.

In conclusion, this study demonstrates the initial clinical feasibility of a DTVA system for telesurgery in real-world settings. DTVA demonstrably mitigates latency-induced performance degradation in controlled non-clinical experiments. This experimentally-confirmed capability provides a technical foundation supporting the feasibility of performing complex procedures such as radical nephrectomy under 300 ms communication latency in realistic constraints when utilizing DTVA. By reshaping tolerance to communication latency, the approach extends the operational boundaries of telesurgery. As a foundational technical validation revealing potential operational efficiency gains, this work provides a basis for future research, while achieving clinical maturity necessitates substantial further validation across diverse scenarios and larger cohorts.

## Methods

### Parametric modeling and virtual endoscopic visualization

**Fig. 10:**
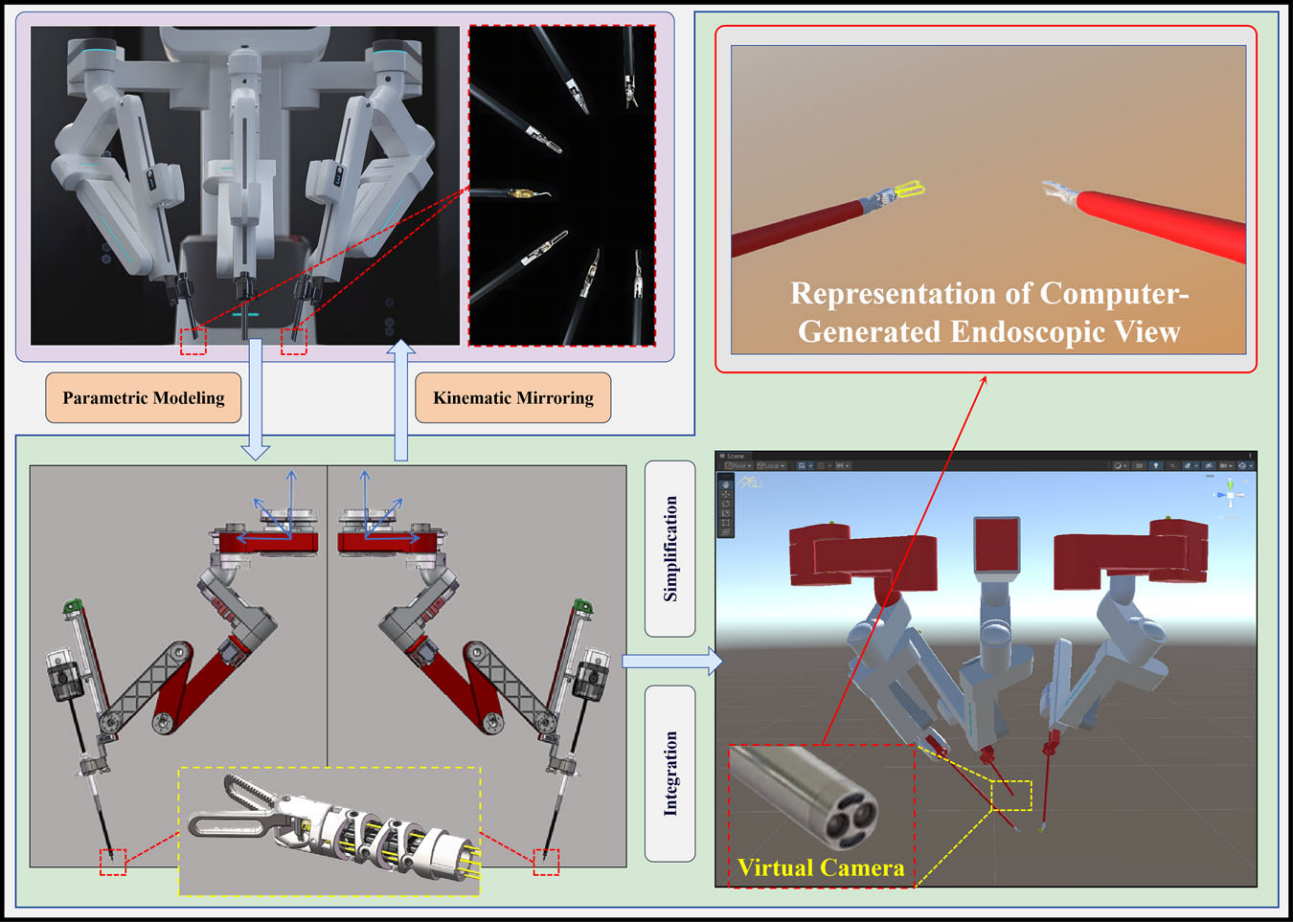
Parametric modeling and virtual endoscope calibration. This study developed a laparoscopic robotic digital twin system using parametric modeling (SolidWorks) and kinematic mirroring. Core components include instruments, endoscope, and robotic arms. Optimized 3D models were integrated into Unity (FBX/HingeJoint/C#) with virtual endoscopic alignment via Zhang’s calibration (intrinsic/extrinsic matrices), ensuring virtual-physical geometric consistency.

This study developed a multi-module digital twin system for laparoscopic surgical robots through parametric modeling and kinematic mirroring. The system architecture encompasses three core components: surgical end-effectors, an endoscope, and robotic arms. Parametric 3D models were constructed in SolidWorks Premium 2024 (Dassault Systèmes SolidWorks Corporation, Waltham, MA, USA) and optimized using mesh simplification algorithms that preserve critical motion joints and functional surfaces while ensuring real-time interactivity. These models were imported into the Unity 2022 (Unity Technologies, San Francisco, CA, USA) engine via Filmbox (FBX) format, where HingeJoint components established instrument degrees of freedom, with joint angle constraints and synchronized control implemented through C# scripting. For endoscopic visualization, a virtual camera was optically parameterized to replicate the geometric and optical specifications of the physical endoscope. Critical parameters, including intrinsic/extrinsic matrices and distortion coefficients derived from Zhang’s calibration framework, were integrated to intrinsically correct radial and tangential distortions, ensuring accurate geometric alignment between the virtual and physical environments. (Fig. 10).

### Hierarchical digital twin architecture for real-time physical-virtual synchronization

**Fig. 11:**
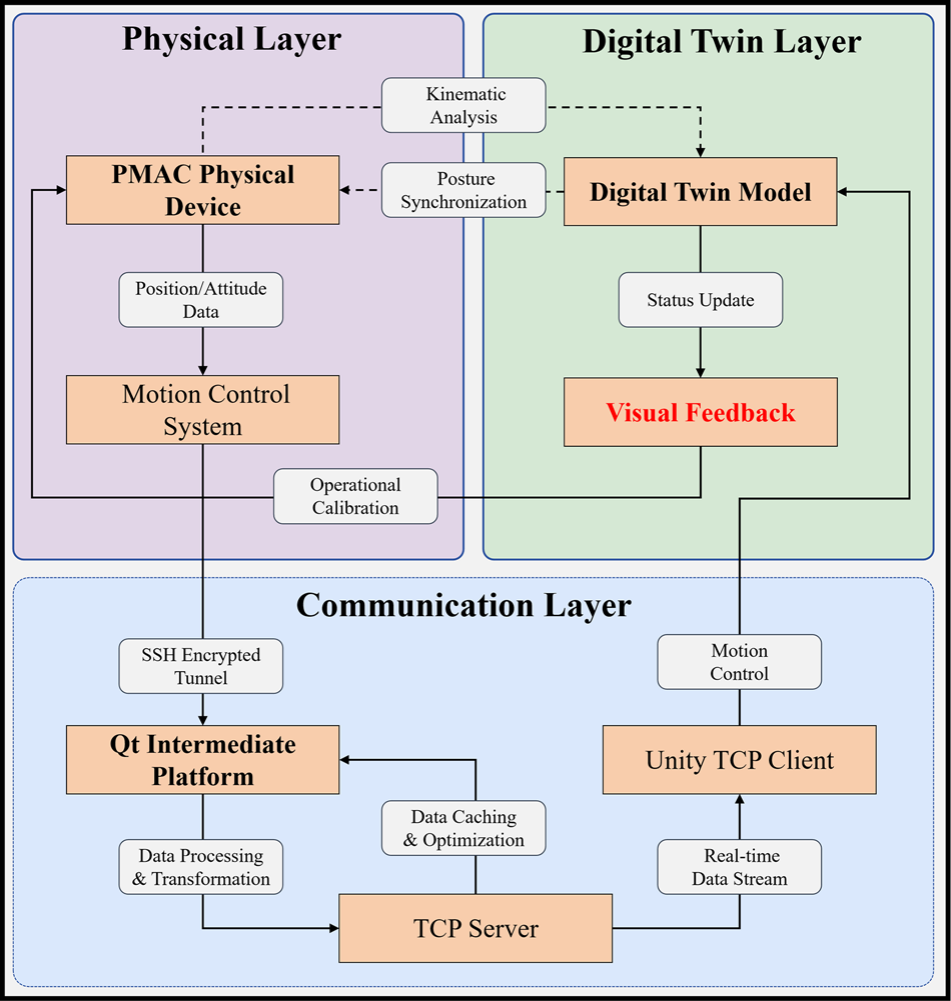
Tri-layered digital twin framework for real-time physical-virtual synchronization via coordinated operations. This framework enables real-time data exchange between physical instruments and virtual models. The physical layer (PMAC) acquires system parameters, transmitted via SSH to a Qt-based middleware, then to Unity via TCP for dynamic virtual model updates. Ensures closed-loop synchronization across acquisition, conversion, and adaptive feedback.

This study presents a tri-layered digital twin framework facilitating data synchronization and state interaction between surgical instruments and their virtual counterparts through coordinated operations (Fig. 11). The physical layer revolves around a Programmable Multi-Axis Controller (PMAC) acquiring the target pose matrix of surgical instruments in real-time. Each numerical element constituting this matrix is defined as a positional P-parameter, collectively representing the spatial state (position and orientation) of the instrument. These parameters are securely transmitting data via Secure Shell (SSH) protocol to a Qt-based middleware platform (The Qt Company, Espoo, Finland) in the communication layer for protocol parsing and format conversion. A stable communication channel using Transmission Control Protocol (TCP) is then established between the intermediate layer and the Unity-based digital twin layer, enabling bidirectional data flow. The communication layer facilitates cross-protocol data adaptation through standardized industrial interfaces, ensuring bidirectional mapping between physical device parameters and virtual model states. Meanwhile, the digital twin layer employs the Unity engine to dynamically update virtual model states via visual feedback mechanisms, allowing surgeons to adjust their operative actions dynamically based on the visual feedback from the twinned instruments. It establishes a closed-loop technical chain that includes “data acquisition, protocol conversion, virtual-physical synchronization, and adaptive feedback”.

### Kinematic modeling and inverse kinematics resolution under RCM constraints

For motion simulation of virtual manipulators and surgical instruments, joint angles are determined through inverse kinematics resolution. In modeling the Remote Center of Motion (RCM) manipulator kinematics, we established its forward kinematics using a modified Denavit-Hartenberg (D-H) method, enabling calculation of the manipulator’s pose matrix *T*_*1*_.

Considering the RCM constraint, our system employs a local coordinate system-based approach {*O*_RCM_} for joint angle computation was introduced relative to the manipulator base frame {*O*_*0*_}.

This method simplifies parameter calculation by directly solving motion parameters for two rotational joints and one prismatic joint within the RCM local coordinate system. The rotational joints consist of a roll joint (*JPos*1) and a pitch joint (*JPos2*), while the prismatic joint (*JPos*3) controls the axial motion of surgical instruments.

The RCM local coordinate system {*O*_RCM_} is defined relative to the manipulator base coordinate system {*O*_*0*_}, with positional offsets determined by three-axis displacements in the base coordinate system. Its rotational orientation involves an inclination angle $\theta$, which depends on initial installation parameters of the robotic arm.

For any target point P (*x*_*0*_*, y*_*0*_*, z*_*0*_) in the base coordinate system {*O*_*0*_}, its coordinates (*x*_RCM_*, y*_RCM_*, z*_RCM_) in {O_RCM_} can be derived through:

1 $$\left[\begin{array}{l}{x}_{\mathrm{RCM}}\\ {y}_{\mathrm{RCM}}\\ {z}_{\mathrm{RCM}}\end{array}\right]={R}_{x}\left(\theta \right)\left[\begin{array}{l}x\\ y-\Delta y\\ z-\Delta z\end{array}\right]{\mathrm{where}}{\,R}_{x}\left(\theta \right)=\left[\begin{array}{rcl}1 & 0 & 0\\ 0 & \cos \theta & -\sin \theta \\ 0 & \sin \theta & \cos \theta \end{array}\right]$$

where $\Delta$*x*, $\Delta$*y*, $\Delta$*z* represent axial offsets between coordinate systems.

The joint-space motion parameters (*JPosN*) are then calculated as:

2 $${\rm{J}}=\left[\begin{array}{l}{{Jpos}}_{1}\\ {{Jpos}}_{2}\\ {{Jpos}}_{3}\end{array}\right]=\left[\begin{array}{l}\arctan 2({x}_{\mathrm{RCM}},\sqrt{{{x}_{\mathrm{RCM}}}^{2}+{{z}_{\mathrm{RCM}}}^{2}})\\ -\arcsin \frac{{y}_{\mathrm{RCM}}}{\sqrt{{{x}_{\mathrm{RCM}}}^{2}+{{y}_{\mathrm{RCM}}}^{2}+{{z}_{\mathrm{RCM}}}^{2}}}\\ \sqrt{{{x}_{\mathrm{RCM}}}^{2}+{{y}_{\mathrm{RCM}}}^{2}+{{z}_{\mathrm{RCM}}}^{2}}\end{array}\right]$$

Instrument joint parameters are determined through resolution of the instrument pose matrix *T*_*2*_, calculated by:

3 $${T}_{2}={T}_{1}^{-1}\cdot T={T}_{1}^{T}\cdot \,T$$

where *T* represents the system-defined final pose matrix and *T*_*1*_ is derived from manipulator forward kinematics.

Each element *IRot*_*ij*_ in *T*_*2*_ is computed through:

4 $${{IRot}}_{{ij}}={\sum }_{k=1}^{3}{{ARot}}_{{ki}}\cdot {p}_{{kj}}$$

where *p*_*ij*_ denotes elements in matrix *T* and *ARot*_*i*j_ corresponds to elements in matrix *T*_*1*._

The instrument orientation involves three sequential rotations:

5 $$\left[\begin{array}{l}{\beta }_{1}\\ {\beta }_{2}\\ {\beta }_{3}\end{array}\right]=\left[\begin{array}{l}\arctan 2\left(\frac{{{IRot}}_{12}}{{{IRot}}_{22}}\right)\\ \arcsin \left({{IRot}}_{32}\right)\\ \arctan 2\left(\frac{{{IRot}}_{31}}{{{IRot}}_{33}}\right)\end{array}\right]$$

Where *β*_*1*_ is the roll angle, *β*_*2*_ is the pitch angle and *β*_*3*_ is the yaw angle.

**Fig. 12:**
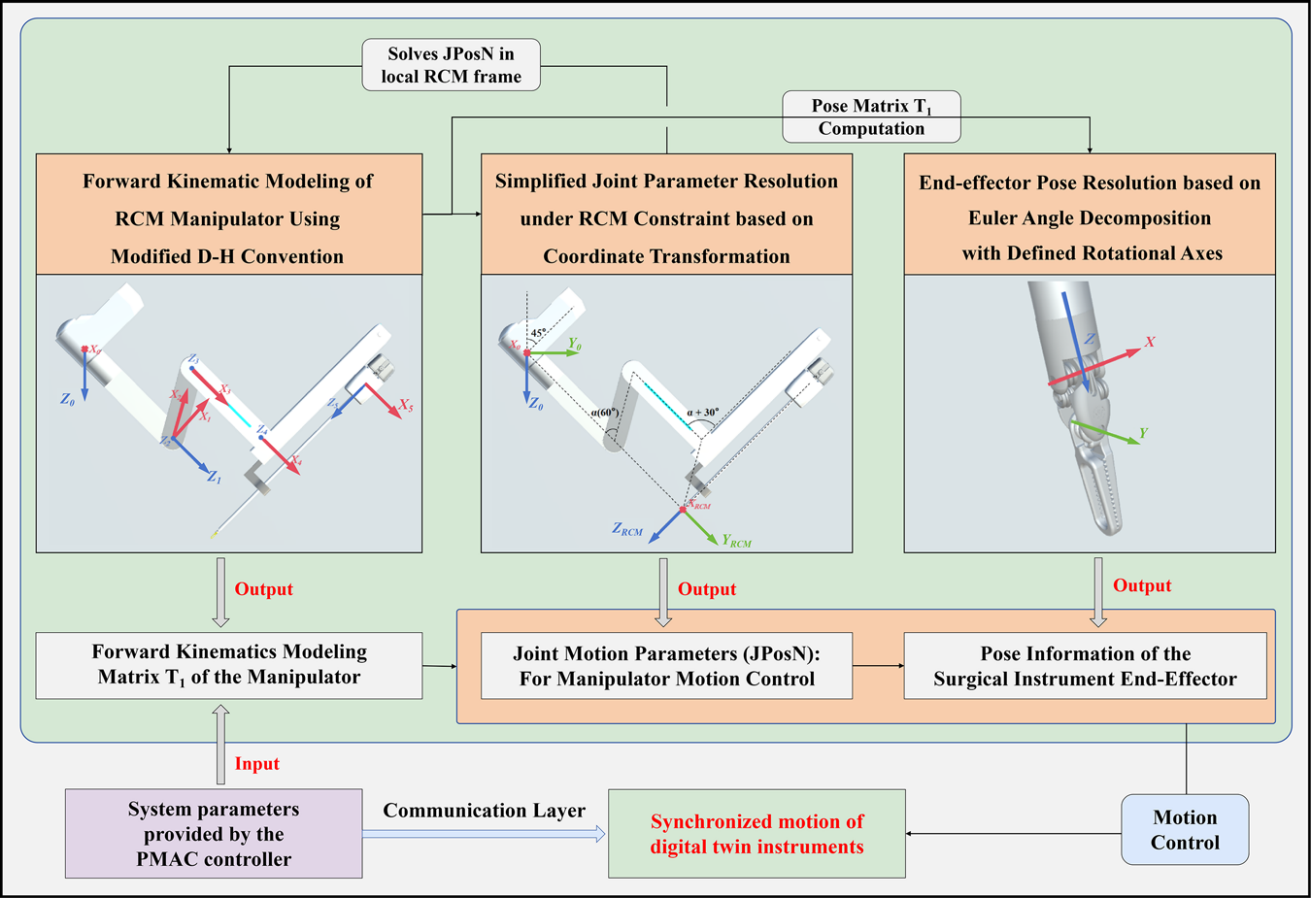
Real-time Master-Slave motion synchronization for surgical digital twins. The workflow for achieving motion synchronization between physical surgical instruments and their digital twin counterparts. Raw PMAC controller data, encoding real-time joint states of the physical manipulator, are processed through the proposed forward and inverse kinematics resolution framework. The RCM-constrained local coordinate system enables direct computation of rotational (roll, pitch) and prismatic joint parameters (JPos_1-3_), while the instrument pose matrix (T_2_) resolves orientation angles (β_1_-β_3_) via sequential rotations. These derived motion parameters are dynamically mapped to the Unity-based digital twin model, ensuring geometric correspondence and real-time positional alignment between virtual and physical instruments.

This kinematic framework enables precise resolution of motion parameters for both RCM manipulator and surgical instruments based on raw data from physical controllers, ensuring accurate master-slave synchronization between the digital twin model in Unity3D and actual surgical devices. (Fig. 12)*.*

### Integrated real-time field-of-view fusion

**Fig. 13:**
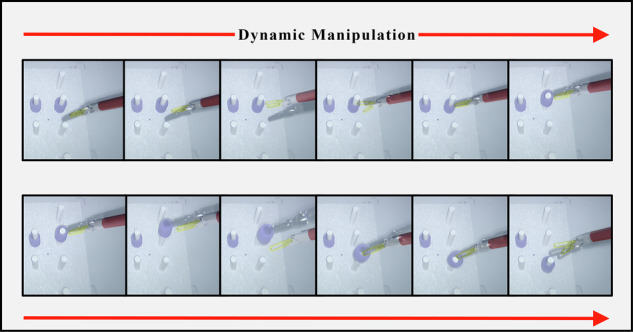
Integrated real-time field-of-view fusion interface combining digital twin and endoscopic video for surgical feedback. This study implements real-time field-of-view fusion by superimposing endoscopic video (captured via Unity’s WebCamTexture/OpenCV) and digital twin renders (Unity RenderTexture) using OpenCV’s weighted blending (adjustable blendFactor).

Based on the real-time field of view fusion method combining the digital twin model and camera video streams, the surgeon’s visual perception during remote operations is enhanced by weighted superposition of the actual endoscope footage and the digital twin environment footage. The real-time camera footage is captured using Unity’s WebCamTexture and subsequently converted into the Matrix (Mat) format provided by Open Source Computer Vision Library (OpenCV). Similarly, the digital twin footage, rendered through RenderTexture, is also converted into Mat format. The weighted superposition function in OpenCV is utilized to generate the fused footage, with the blendFactor parameter allowing adjustable weight distribution between the two. The final fused footage is converted into Unity texture format in real-time and displayed on the RawImage component. This fused interface enables simultaneous monitoring of both physical instrument motion (via endoscopic video) and virtual model dynamics, ensuring intuitive alignment between the surgeon’s actions and the digital twin’s feedback (Fig. 13).

### Configuration of surgical robot system and network communication

The proposed DTVA system framework was implemented on the MicroHand S surgical robot^®^ (WEGO Surgical Robot Company, Weihai, China), establishing a geographically distributed master-slave architecture with the master console in Weihai and slave manipulators in Qingdao (209.2 km apart). The surgical robot system possesses an inherent baseline latency of approximately 150 ms^23^, present in all operational modes (local or remote), arising from its servo control cycle (<1 ms), mechanical response (40 ms), endoscopic image processing (50 ms), and video codec processing (60 ms).

A carrier-grade packet transport network (PTN) dedicated line (China Mobile Communications Group Co., Ltd., Qingdao, China) connected the master and slave terminals, featuring operator-guaranteed Quality of Service (QoS): 200 Mbps bandwidth, 1500-byte Maximum Transmission Unit (MTU), a baseline network latency of 5 ms, and packet loss rate <0.001%. Programmable deterministic delays were added to this baseline network latency to investigate the impact of communication latency on surgical performance and DTVA efficacy. This was implemented by programmatically extending bidirectional master-slave transmission intervals, introducing controlled queuing delays while preserving other baseline network parameters.

All subsequent validation experiments were conducted within the geographically distributed PTN-based architecture under combined latency conditions. Throughout this study, communication latency denotes the sum of baseline network latency (5 ms) and programmatically added delay, with all reported latency values (e.g., 300 ms, 600 ms) referring exclusively to this communication latency component. Total operational latency constitutes the fixed 150 ms inherent baseline latency plus the specified communication latency. Unless explicitly stated, all reported latency values refer to communication latency only and exclude inherent baseline latency of the surgical robot system.

### DTVA system accuracy quantification

**Fig. 14:**
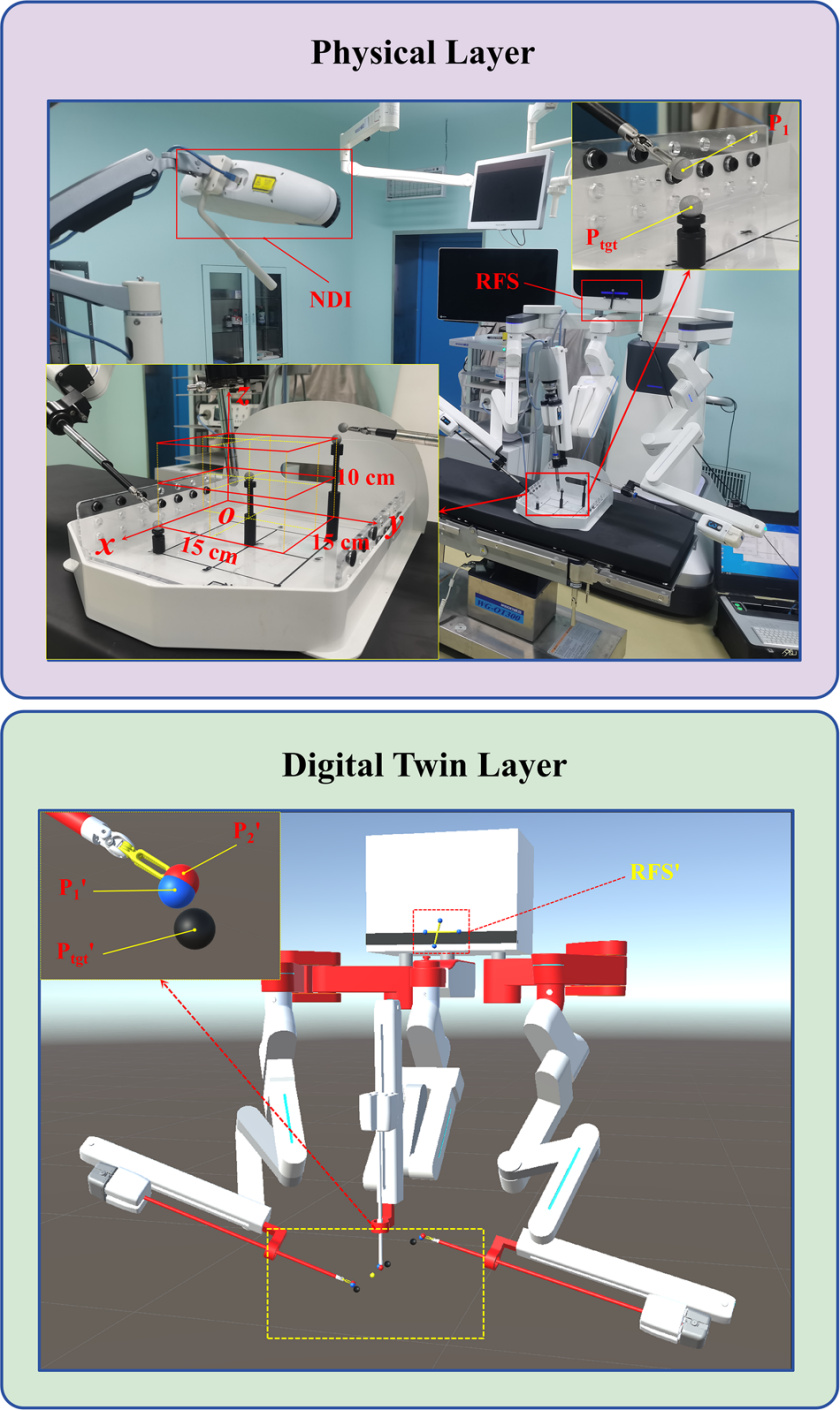
Schematic diagram of NDI-based virtual-physical spatial registration experiment. Physical workspace configuration (Upper): A 15 × 15 cm² two-dimensional grid baseplate with height-adjustable brackets to define spatial boundaries of the operational workspace, featuring fiducial markers (*P*_tgt_) and robotic arms tracking end-effector position (P_1_) via NDI optical system and rigid reference structure (RFS). Digital twin implementation (Lower)**:** Unity-based virtual environment dynamically maps physical coordinates (P_1_’) through spatial transformation matrices, with alignment error (δ_2_ = ‖P_1_’ – P_2_’‖) computed between transformed physical positions (P_1_’) and pre-registered virtual counterparts (P_2_’).

This study established a virtual-physical synchronization framework for surgical robotic end-effectors using a Northern Digital Inc. (NDI, Waterloo, ON, Canada) optical tracking system and digital twin spatial registration (Fig. 14). A physical reference coordinate system (*O-XYZ*) was constructed with an NDI system and rigid reference structure (RFS), while a proportional virtual coordinate system (*O’-X’Y’Z’*) was implemented in Unity. Spatial alignment was achieved by matching four NDI markers on the physical RFS to their virtual counterparts (RFS’). All physical coordinates, acquired via NDI-calibrated marker centroids, were dynamically mapped to the virtual system through the coordinate transformation matrix.

The experimental workspace comprised a 15 × 15 × 10 cm³ layered structure with a 15 × 15 cm² two-dimensional grid baseplate with height-adjustable brackets, defining the spatial boundaries. Nine geometrically representative points (eight cuboidal vertices and centroid) were physically demarcated using NDI reflective spheres, with physical centroid coordinates (*P*_tgt_) mapped to virtual targets (*P*_tgt_’) via coordinate transformation. Three robotic arms (endoscopic and bilateral instrument arms) were fitted with NDI reflective spheres at their end-effectors for validation. In the digital twin environment, virtual spheres (P_2_’) were initialized at registration points (P_reg_’) derived from transformed physical fiducial coordinates (P_1_’), enforcing spatial coincidence (*δ*_*1*_ *= ‖ P*_*1*_*’ – P*_reg_*’ ‖* *=* *0*) during registration. Post-registration, P_2_’ maintained fixed spatial relationships with virtual robotic arms, mirroring their physical counterparts. During experimental trials, P_2_’ dynamically followed the motion of virtual instruments, analogous to how physical markers (P_1_) tracked real robotic arm movements. During experiments, robotic arms were maneuvered to positions surrounding the nine fiducial markers. Upon stabilization, physical spatial coordinates (P_1_) acquired from NDI tracking systems were mapped to their pre-registered virtual coordinates (P_1_’), while virtual sphere coordinates (P_2_’) were directly retrieved from the Unity engine.

Positioning errors were quantified using a Euclidean distance model:

6 $$\delta 2\,=\Vert \,P{1}^{{\prime} }-\,P{2}^{{\prime} }\Vert$$

Thereby evaluating the spatial mapping accuracy of the DTVA system. Triplicate measurements were conducted per robotic arm at distinct orientations near each fiducial marker. Notably, this NDI-based registration served solely to quantify static spatial deviations in this validation study.

### DTVA system latency quantification

The intrinsic latency of the hierarchical DTVA framework is defined as the time interval between the reception of data transmitted from the PMAC controller by the Qt and the completion of the fused visual display based on that dataset. This intrinsic latency consists of two components. The first component (L₁) represents the time interval from when the data are received by Qt, processed, and then transmitted to the Unity. The second component (L₂) refers to the interval from the moment Unity receives the data to the final rendering of the fused display image.

Within the Qt framework, received PMAC data is assigned a unique frame identifier (dataFrameId). Qt’ s high-precision timing mechanism (QElapsedTimer) then timestamps both data reception (startTicks_1_) and transmission (endTicks_1_), enabling calculation:

7 $${{\rm{L}}}_{1}=\,{{\rm{endTicks}}}_{1}-\,{{\rm{startTicks}}}_{1}$$

In Unity, a high-precision timing architecture using System.Diagnostics.Stopwatch with CPU timestamp counters (TSC) achieved 100-nanosecond resolution. When the data sent by Qt is received, the start state (startTicks_2_) is recorded, and the completion state is detected by the Unity event trigger from binding to image display completion (endTicks_2_), enabling calculation:

8 $${{\rm{L}}}_{2}\,=\,\frac{({{\rm{endTicks}}}_{2}-{{\rm{startTicks}}}_{2})\,\times {10}^{6}}{{\rm{Stopwatch}}.{\rm{Frequency}}}$$

A dataset of 200 consecutive frames was captured under hardware-synchronized conditions to statistically characterize L₁ and L₂, with cross-domain timestamp correlations rigorously maintained between physical data streams and virtual state transitions.

### Experimental evaluation of the DTVA system in teleoperated peg transfer task

**Fig. 15:**
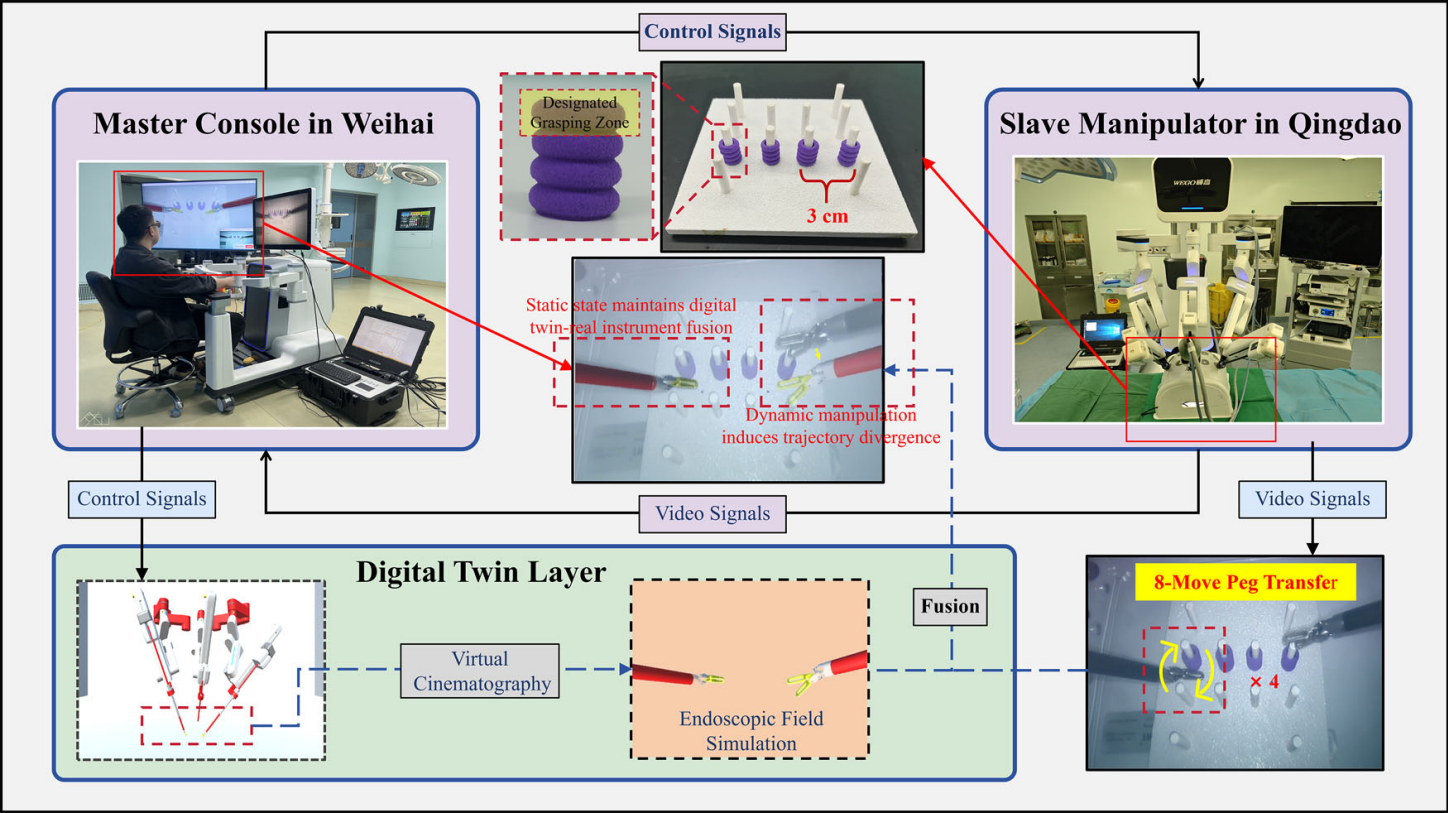
Teleoperated laparoscopic peg transfer task design incorporating digital twin visual assistance (DTVA) across different latency conditions. The Surgeon console with fused visual feedback was located in Weihai, while the slave robotic arm was situated in Qingdao, connected via communication latency regimes. Three surgeons transferred pegs using single-handed operation, with time constraints and specific grasping rules. Prior to each task, comprehensive initialization adjustments and PMAC-based spatial calibration were conducted to ensure alignment between the physical slave arm and its virtual counterpart, with the endoscopic arm remaining stationary throughout the task to maintain spatial reference integrity.

This study utilized a standardized laparoscopic peg transfer task to assess surgical performance across four defined communication latency regimes (5 ms, 300 ms, 600 ms, 900 ms), with experimental groups operating both with and without DTVA. Three experienced surgeons, who had surpassed the learning curve for robotic surgery, were recruited. Participants were required to transfer four horizontally aligned pegs sequentially from upper to lower sockets (spaced 3 cm apart between adjacent pairs) on a restricted pegboard and return them to their original positions. The task mandated single-handed operation: the left hand for the two left-sided pegs and the right hand for the two right-sided pegs. Grasping was restricted to the topmost end of each peg, and no external support from the pegboard or pegs was permitted. Each successful peg transfer represented 12.5% of total task progress (eight transfers required for 100% completion). The predefined task duration was set at 150 s, and the completion rate was quantified as the percentage of progress achieved within this temporal constraint. When transfers remained incomplete at 150 s, the task continued until full completion, with the extended time recorded accordingly. (Fig. 15, Supplementary Video 1).

To ensure operational uniformity across experiments, comprehensive initialization adjustments and system verifications were performed for both physical and virtual robotic arms prior to each procedure. This involved precise calibration of initial positions between the physical slave arm and its virtual counterpart through implementation of P-parameters in the PMAC of the slave system’s lower-level architecture, with the endoscopic arm remaining stationary throughout subsequent operational phases to maintain spatial reference integrity. Each of the 3 surgeons conducted three repetitions under each experimental condition, with randomized task sequences and a minimum 3-h interval between tasks to minimize potential practice effects.

Task execution was quantified using five metrics: task completion rate, task completion time, total trajectory lengths of bilateral instrument end-effectors, average velocity, and operator workload. Total trajectory lengths were determined within the virtual coordinate system (*O’-X’Y’Z’*) by acquiring 3D coordinates of non-visible markers fixed to virtual instrument tips through Unity engine tracking. Average velocity was derived by dividing total trajectory length by the recorded task completion time.

Operator workload was assessed using a modified NASA Task Load Index (NASA-TLX) scale through three Delphi consensus rounds with 5 surgical experts, which evaluated six dimensions: mental demand, physical demand, temporal demand, effort, performance, and frustration^24,25^. Each dimension was scored on a standardized 10-point scale with detailed behavioral descriptors and operational perceptions provided for each score level to ensure consistent interpretation across participants. Specifically, the scale was anchored at four critical points (1, 4, 7, and 10) to represent distinct workload states, ranging from effortless operation to task failure. Pre-assessment standardized instructions clarified scoring criteria to ensure inter-rater consistency. To capture contemporaneous cognitive states, workload scores were collected immediately following the first task completion under each unique condition (Latency conditions ± DTVA usage).

### Experimental evaluation of the DTVA system in teleoperated suturing task

**Fig. 16:**
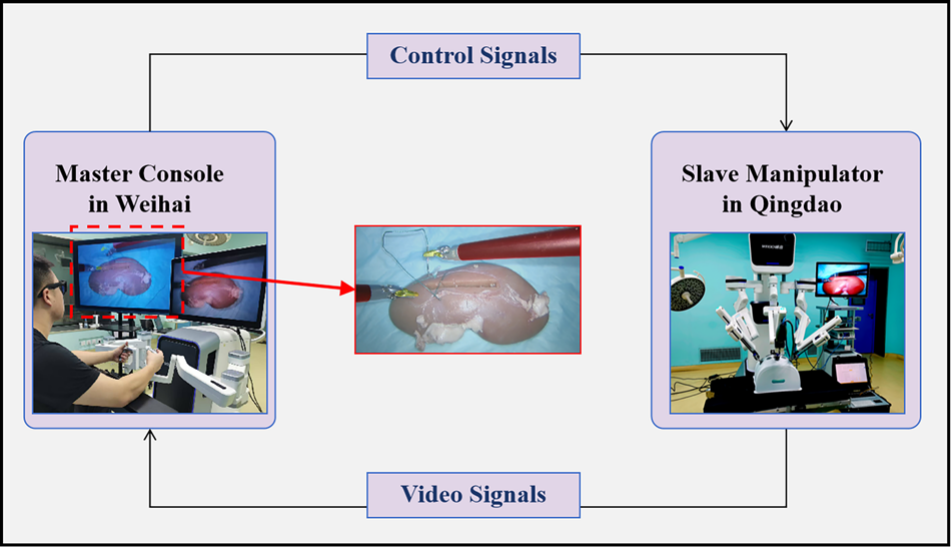
Teleoperated laparoscopic suturing task design incorporating digital twin visual assistance (DTVA) across different latency conditions. This study evaluated the Digital Twin Visual Assistance (DTVA) system’s effectiveness for suturing under communication latency (5, 300, 600, 900 ms). A surgeon performed five simple continuous sutures on standardized wedge-shaped incisions (5 × 0.5 × 1.5 cm) in ex vivo porcine kidneys under each latency condition, both with and without DTVA.

To evaluate the effectiveness of the proposed DTVA system for suturing under communication latency, suturing tasks were performed on ex vivo porcine renal tissue (Fig. 16). Experiments were conducted across four communication latency conditions (5 ms, 300 ms, 600 ms, 900 ms), with performance compared both with and without DTVA assistance. A standardized wedge-shaped incision (5 cm length × 0.5 cm width × 1.5 cm depth) was created on the surface of fresh ex vivo porcine kidneys. A single surgeon performed 5 simple continuous sutures using 5-0, 1/2-circle surgical sutures (20 cm length, knotted and marked 20 cm from the needle tail) to close the incision under each latency/DTVA combination. Suturing adhered to core principles: symmetry (entry and exit points equidistant from wound edges), equidistant stitch spacing across the entire wound, and precise edge approximation with appropriate tension (neither too loose nor too tight). The specific suturing path was determined by the surgeon following these principles. If tissue tearing occurred during suturing, the procedure continued until all five sutures were completed. Each experimental condition was repeated three times in a randomized task sequence, with sessions separated by at least three hours to minimize practice effects. Except for the operative task content, all experimental configurations were identical to those of the peg transfer task.

Suturing task performance was quantified using multiple metrics: task completion time, total trajectory lengths (bilateral instrument end-effectors), average velocity, suturing quality, tissue tearing incidence. The measurement methodologies for task completion time, total trajectory lengths and average velocity were identical to those employed in the peg-transfer experiment.

**Fig. 17:**
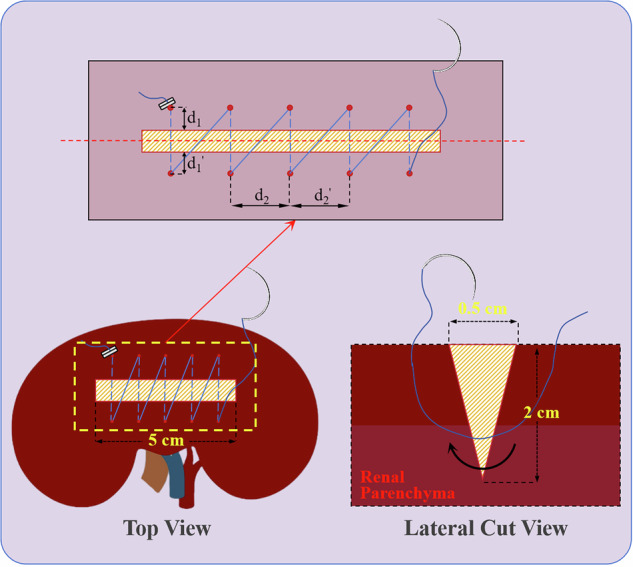
Schematic illustration of sutured renal incision with key suture quality parameters. Schematic depicting the standardized wedge-shaped renal incision and critical suture quality metrics.

Suture quality was assessed through a parametric framework evaluating three components: stitch symmetry (Q₁), suture spacing uniformity (Q₂), and tissue edge apposition (Q₃). The symmetry parameter Q₁ was defined as the maximum absolute difference in perpendicular distances from needle entry/exit points to the wound edges (‖d₁ - d₁’‖_max_). The uniformity parameter Q₂ was determined by the maximum interstitch distance variation (‖d₂ - d₂’‖_max_). Q₃ was qualitatively evaluated based on tissue alignment integrity. Each component was scored on a 3-point scale: 3 points for ‖d₁ - d₁’‖_max_ ≤ 2 mm, ‖d₂ - d₂’‖_max_ ≤ 2 mm, or precise approximation without deformities; 2 points for deviations of 2–5 mm or minor misalignment; 1 point for deviations >5 mm or extensive defects. The composite score Q_c_ was calculated as the sum (Q₁ + Q₂ + Q₃). A critical penalty clause mandated automatic disqualification (Q_c_ = 0) for any tissue tearing; the incidence of such tears was additionally recorded as a safety parameter (Q_s_). This framework enabled a standardized quantitative assessment of performance. (Fig. 17)*.*

### Clinical assessment of the DTVA system in telesurgery

The study was registered on the clinical trials website (https://clinicaltrials.gov/) with the registration number NCT05739812 and on the Chinese Clinical Trial Registry (http://www.chictr.org.cn) with the registration number ChiCTR2400089700. It was also reviewed and approved by the Academic Committee and Ethics Committee of The Affiliated Hospital of Qingdao University (Approval Number: QYFYEC2024-71). All study activities were conducted in accordance with the Declaration of Helsinki and the Code of Practice for the Management of Clinical Trials of Medical Devices. Patients 18 years of age or older who were scheduled for radical nephrectomy were eligible for enrollment (Further details of the Inclusion/Exclusion criteria are provided in the Supplementary Table 2). All participants were informed about the unique aspects of this approach and provided written informed consent specifically for the telesurgery.

**Fig. 18:**
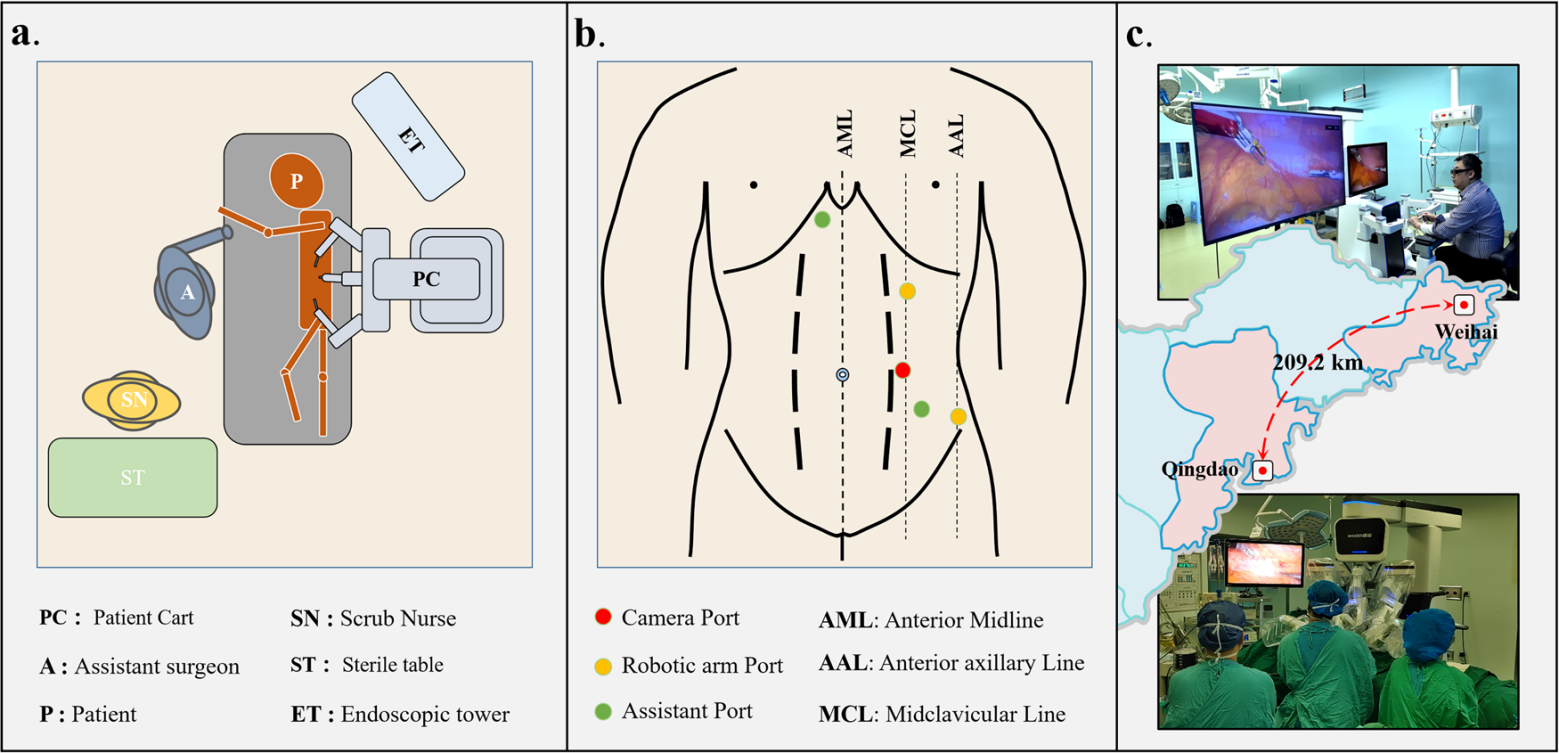
Clinical trial configuration for DTVA telesurgical nephrectomy. **a** Operating Room Layout. This is the layout of the operating room when the tumor is located in the right kidney; if the tumor is located on the left side, the patient is placed in the left lateral position, and the position of the assistant, the robotic arms and the monitor are switched to the opposite side. **b** Trocar Placement. For the Light-side procedure, the camera port was placed at the lateral margin of the rectus abdominis muscle at the level of the umbilicus; the ports of the two robotic manipulator arms were located at the subcostal and anterior axillary lines in the midclavicular line, respectively, at an angle of approximately 110 from the camera ports; the auxiliary port was chosen to be between the camera port and the robotic manipulator holes (generally chosen on the right side of the assistant); For the right-side procedure, an additional 5-mm auxiliary port was added to pick apart the liver to expose the surgical region, which is positioned under the xiphoid process. **c** Master-slave system configuration of the DTVA telesurgery platform. Master side in Weihai: Surgeon console with fused visual feedback (real endoscopic view augmented with virtual instrument overlays). Slave side in Qingdao: Patient-side robotic arms executing teleoperated maneuvers.

With patient safety as the paramount consideration, the communication latency threshold was conservatively set to 300 ms for clinical validation, with no higher delay conditions tested. All procedures under this latency condition exclusively utilized the DTVA System without establishing control groups. A single experienced urologist performed all teleoperations, executing critical maneuvers including delicate dissection, cauterization, and sectioning through the DTVA interface. Each endoscopic arm adjustment initiated a spatial calibration process, where the virtual instrument’s position was calibrated in relation to the physical instrument’s position within the endoscopic view, aligning the physical-virtual instrument pairs within the fused visual field. To further ensure patient safety, a urological surgical assistant with extensive experience in robotic, laparoscopic, and open surgeries was present. In the event of any failure during the remote surgery, the assistant was capable of taking over and completing the procedure. Clinical data were collected and recorded by dedicated personnel. (Fig. 18).

The primary endpoints were feasibility (procedure completion via exclusive teleoperation without conversion) and safety (Clavien-Dindo grade <III complications^26^). Any event outside these definitions of feasibility and safety was considered a failure of the remote surgery. Secondary endpoints included the duration of the remote surgery, blood loss, incidence of adverse events, postoperative 24-h Visual Analog Scale (VAS) pain scores^27^, and follow-up outcomes at 30 days and postoperatively.

### Statistical methods

SPSS Statistics software (version 25.0; IBM Corp, Armonk, NY, USA) was used for all statistical analyses. Quantitative variables are expressed as mean ± standard deviation (SD) or median (range) where appropriate. Categorical data are presented as frequencies with percentages. Pearson’s correlation coefficient was used to assess the relationship between virtual-real spatial mismatches (δ_2_) and registration fiducial displacements (δ_1_). To examine the interaction and main effects of the independent variables, a two-factor ANOVA (communication latency × DTVA use) followed by simple effects analysis performed using Bonferroni-adjusted pairwise comparisons was performed to analyze the effects. A P-value of less than 0.05 was considered statistically significant. Data visualization was performed using Origin 2024 (OriginLab Corporation, Northampton, MA, USA).

## Supplementary information

Supplementary information

Supplementary Video1

## Data Availability

The data generated and analyzed during this study are available from the corresponding author upon reasonable request.

## Code availability

The code used during this study is available from the corresponding author upon reasonable request.
