# Supplementary material for: Enhancing telesurgical safety with predictive digital twin synchronization: a framework for latency compensation in robotic surgery
Source: NPJ Digit Med. 2026 Jan 13;9:108. doi: 10.1038/s41746-025-02283-w (PMC12864800; doi:10.1038/s41746-025-02283-w)
Supplement: Supplementary file 1 — Supplementary information [file 41746_2025_2283_MOESM1_ESM.pdf]

Supplementary Material

This PDF file includes:

- Supplementary Table 1. Modified NASA Task Load Index (NASA-TLX) scale.
- Supplementary Table 2. Detailed Patients Selection Criteria for the Study.
- Supplementary Video 1. DTVA-Enhanced Teleoperated Peg Transfer task under Latency Conditions

Note: Items above follow their sequential order of appearance in the main text.

Supplementary Table 1. Modified NASA Task Load Index (NASA-TLX) scale

Dimension	Description		Score
Mental Demand	1	No active thinking; instinctive, relaxed, and smooth operation.	
	4	Occasional focus needed; tasks completed without strategy adjustment.	
	7	Sustained concentration; active rhythm adjustment and predictive actions; increased cognitive load.	
	10	High tension; complex decisions (e.g., repeated corrections); significant mental fatigue.	
Physical Demand	1	Natural hand movements; effortless control; no muscle tension or fatigue; stable operation.	
	4	Slight muscle tension; moderate grip increase; movement continuity unaffected.	
	7	Deliberate effort to maintain stability; hand stiffness; compromised movement continuity.	
	10	Local muscle soreness; frequent posture adjustments or task interruption.	
Temporal Demand	1	Ample time; self-controlled rhythm.	
	4	Occasional need to speed up; slight catch-up; overall rhythm controllable.	
	7	Active time compression; significantly increased time pressure.	
	10	Complete loss of time control; severe task progression lag.	
Effort	1	Effortless; natural operation completion.	
	4	Slight energy expenditure; occasional path adjustments; overall ease.	
	7	Active strategy optimization (e.g., task segmentation); significantly increased energy input.	
	10	Repeated attempts; task difficulty; external assistance or partial abandonment needed.	
Performance	1	Complete precision; no errors (e.g., successful on first attempt).	
	4	Occasional minor deviations; self-correctable.	
	7	Noticeable errors; additional correction steps required.	
	10	Frequent failures; multiple operation repetitions needed.	
Frustration	1	Completely calm; full confidence; no emotional fluctuations.	
	4	Occasional impatience; quickly adjustable.	
	7	Intermittent frustration; active emotional regulation required.	
	10	Extreme frustration; thoughts of task abandonment.	
Was DTVA utilized? Yes □ / Not □	The Communication Latency set during operation: ____ ms		

Supplementary Table 2. Detailed Patients Selection Criteria for the Study

Inclusion Criteria :

- Patients with renal tumors that scheduled radical nephrectomy.
- 18 years of age or older, male or female.
- Body mass index(BMI) 18-30 kg/m^2.
- American Society of Anesthesiologists (ASA) Level I, II or III.
- Subjects (or their legal representatives/guardians) voluntarily participate in the clinical study and have signed the informed consent form.

Exclusion Criteria :

- Participation in any other clinical trial or clinical study within 30 days prior to signing the informed consent form.
- Pregnant or lactating women.
- Have a history of epilepsy or psychiatric disorders.
- Have a history of previous surgery at the target site of this procedure.
- Severe cardiac, pulmonary, hepatic or renal insufficiency, who cannot tolerate the procedure after preoperative evaluation.
- Severe allergies and suspected or established alcohol, drug or drug addiction.
- Patients with abdominal infections, peritonitis, diaphragmatic hernia; severe urinary tract or systemic infections.
- Other conditions that the investigator considers inappropriate for participation in this clinical study.

Supplementary Video 1. DTVA-Enhanced Teleoperated Peg Transfer task under Latency Conditions

This video demonstrates the experimental setup and a representative trial of the peg transfer task under latency conditions. The improved precision and stability afforded by the DTVA in mitigating the effects of latency are visually apparent.
